# Sarcoehrenbergilides D–F: cytotoxic cembrene diterpenoids from the soft coral *Sarcophyton ehrenbergi*†

**DOI:** 10.1039/c9ra04158c

**Published:** 2019-08-29

**Authors:** Mohamed-Elamir F. Hegazy, Tarik A. Mohamed, Abdelsamed I. Elshamy, Ahmed R. Hamed, Mahmoud A. A. Ibrahim, Shinji Ohta, Akemi Umeyama, Paul W. Paré, Thomas Efferth

**Affiliations:** Department of Pharmaceutical Biology, Institute of Pharmacy and Biochemistry, Johannes Gutenberg University Staudinger Weg 5 55128 Mainz Germany; Chemistry of Medicinal Plants Department, National Research Centre El-Tahrir St., Dokki Giza 12622 Egypt elamir77@live.com tarik.nrc83@yahoo.com n1ragab2004@yahoo.com; Natural Compound Chemistry Department, National Research Centre El-Tahrir St., Dokki Giza 12622 Egypt elshamynrc@yahoo.com; Faculty of Pharmaceutical Sciences, Tokushima Bunri University Yamashiro-cho Tokushima 770-8514 Japan umeyama@ph.bunri-u.ac.jp; Computational Chemistry Laboratory, Chemistry Department, Faculty of Science, Minia University Minia 61519 Egypt m.ibrahim@compchem.net; Graduate School of Biosphere Science, Hiroshima University 1-7-1 Kagamiyama Higashi-Hiroshima 739-8521 Japan ohta@hiroshima-u.ac.jp; Department of Chemistry and Biochemistry, Texas Tech University Lubbock TX 79409 USA

## Abstract

A solvent extract of the soft coral *Sarcophyton ehrenbergi* afforded cembrene diterpenoids, sarcoehrenbergilid D–F (1–3). Chemical structures were established by modern spectroscopic techniques with absolute stereochemistries determined by circular dichroism (CD) and time-dependent density functional theory electronic CD calculations (TDDFT-ECD). Cytotoxicity activities for 1–3 were evaluated against three human cancer cell lines: lung (A549), colon (Caco-2) and liver (HepG2).

## Abstract 2

A solvent extract of the soft coral *Sarcophyton ehrenbergi* afforded cembrene diterpenoids, sarcoehrenbergilid D–F (1–3).

## Graphical abstract

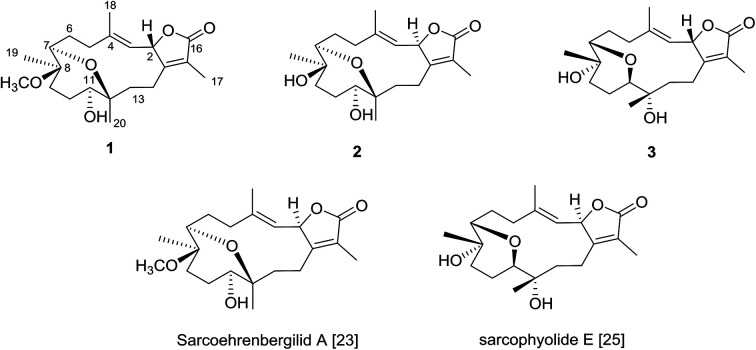

## 1. Introduction

Soft coral of the genus *Sarcophyton* (subclass Octocorallia; order Alcyonaceae; family Alcyoniidae) contain a diversity of cyclic diterpenes that usually contain ethers, lactones or furanes around a cembrane framework.^1,2^ These cembrane diterpenoids exhibit a wide range of structural diversity and biological activity.^3–10^ Cembranoids, the main metabolites identified in the genus *Sarcophyton* have been shown to serve as an effective chemical defense against natural predators of coral.^11^

The leather coral *Sarcophyton ehrenbergi* (von Marenzeller, 1886) produces diverse metabolites with distinct chemical structures as well as promising biological activities.^8,12–17^ Additionally, prostaglandins (PGs) that regulate a broad range of physiological activities, have been isolated from *S. ehrenbergi*.^18,19^

**Fig. 1:**
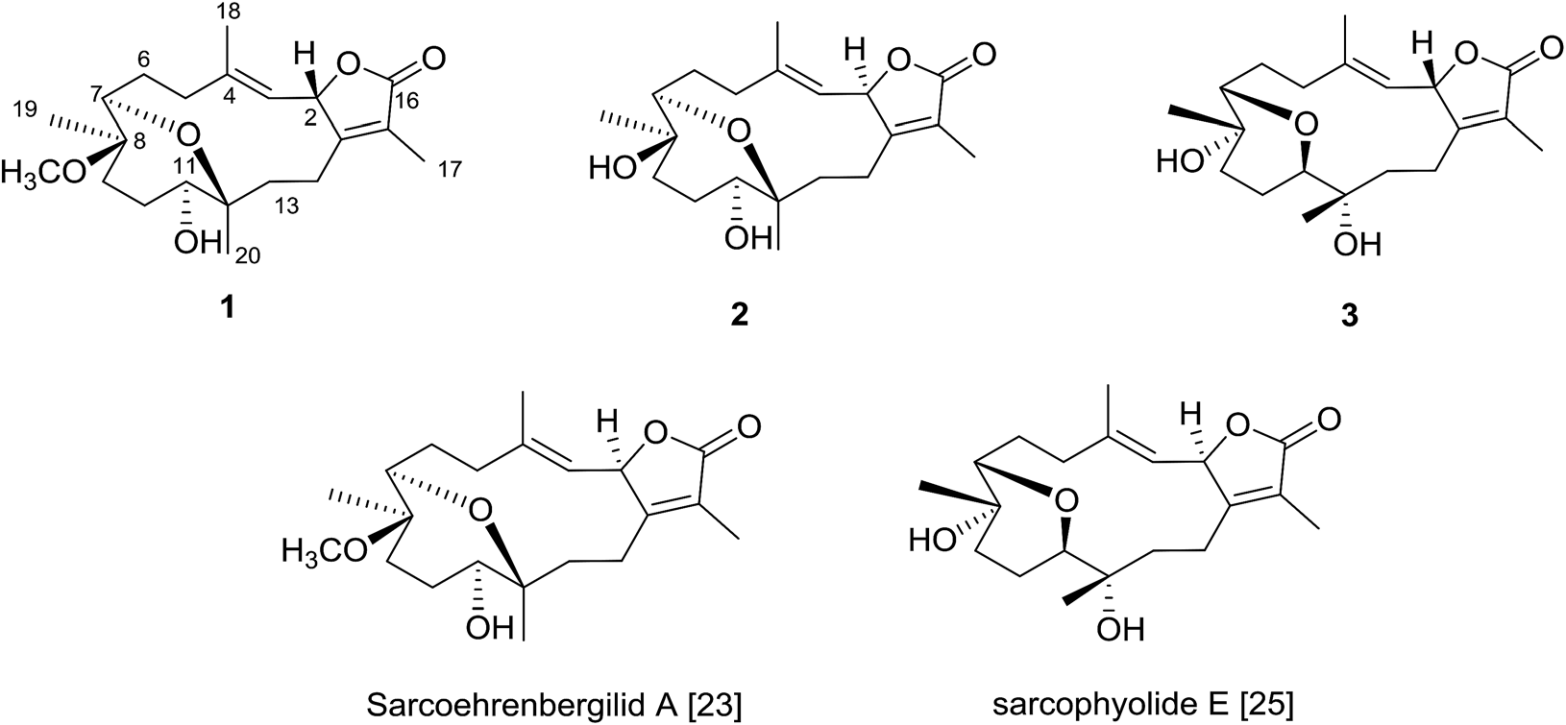
Structures of metabolites 1–3.

The Red Sea contains a high endemic biota including approximately 50 genera of hermatypic soft coral.^20^ While Red Sea marine invertebrates have been historically under-reported within the scientific literature, intensive investigation of Red Sea marine life has occurred over the past ten years.^8,21–23^ To continue efforts to identify new marine metabolites from Red Sea soft coral,^6–8,22–24^ herein we report three cembrene diterpenoids isolated from *S. ehrenbergi* (Fig. 1). Absolute stereochemistry of the newly reported compounds was determined by time-dependent density functional theory-electronic circular dichroism (TDDFT-ECD) calculations. All isolated metabolites were probed against three human cancer cell lines.

## 2. Results and discussion

Freshly collected *S. ehrenbergi* were rapidly frozen by placing in a −20 °C chamber and kept frozen till time of extraction. The chromatographic separation of the methylene chloride : methanol (1 : 1) extract yielded three cembrene diterpenoids derivatives (Fig. 1).

Compound 1 was isolated as a white powder with an optical rotation of [*α*]^25^_D_ +10.1 (*c* 0.02, CHCl_3_). The molecular formula C_21_H_32_O_5_ was determined by high-resolution electron ionization mass spectrum (HREIMS) (*m*/*z* 346.2127 [M − H_2_O]^+^, calcd 346.2149).

**Fig. 2:**
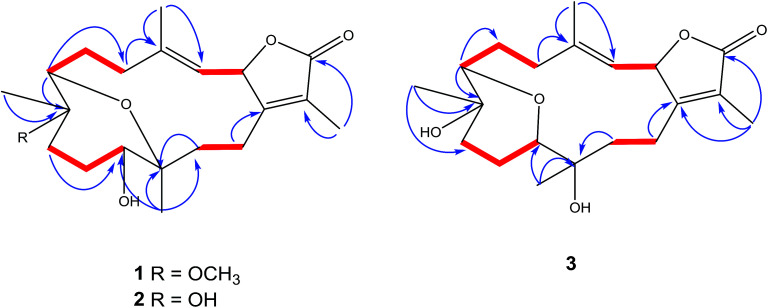
Selected ^1^H–^1^H COSY (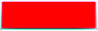) and HMBC (
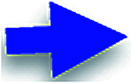
) correlations of 1–3.

The IR spectrum showed absorption bands at *ν*_max_ 3450 cm^−1^ and 1754 cm^−1^ for hydroxyl and keto groups, respectively. The ^13^C NMR and distortion less enhancement by polarization transfer (DEPT) spectrum showed 21 carbon signals, classified as five methyls, six methylenes, four methines and six quaternary carbons (Table 1). Additionally, four oxygenated carbons at *δ*_C_ 76.2 (dC), 78.0 (dC), 78.5 (dC) and 78.1 (sC), four olefinic carbon signals at *δ*_C_ 119.5, 121.8, 147.0 and 163.0. These functionalities were obtained by ^1^H NMR analysis: oxygenated proton signals at *δ*_H_ 3.57 (brd; *J* = 10.0 Hz), *δ*_H_ 3.14 (brd, *J* = 5.0 Hz), *δ*_H_ 5.45 (d; *J* = 10.0 Hz); four methyl singlets at *δ*_H_ 2.02 s, 1.83 s, 1.11 s and 1.03, as well as, one methyl of a methoxy group at *δ*_H_ 3.20 s; olefinic signal at *δ*_H_ 5.14 (d; *J* = 10.0 Hz) signed for a tri-substituted double bond (Table 1). 1D and 2D NMR spectroscopic data comparison (Table 1) closely corresponded to those of previously isolated metabolites from *Sarcophyton* species as well as a previously isolated skeleton by Hegazy *et al.*, 2017 (ref. 5–13, 22 and 23) (Fig. 2).

**Table 1:** ^1^H (500 MHz) and ^13^C (125 MHz) NMR data for compound 1–3^a^ (*δ* in ppm, *J* in Hz)

No.	1^b^		2^c^		3^c^	
	*δ*_H_	*δ*_C_	*δ*_H_	*δ*_C_	*δ*_H_	*δ*_C_
1	—	163.0	—	164.4	—	163.9
2	5.45 d (10.00)	78.1	5.54 d (9.5)	81.0	5.38 brd (10.00)	80.2
3	5.14 d (10.00)	119.5	4.99 d (9.00)	119.4	5.10 brd (10.00)	119.5
4	—	147.0	—	141.6	—	144.4
5	1.87 m, 2.37 brd (14.00)	34.6	2.11 m, 2.37 m	41.0	1.85 m, 1.62 m	37.00
6	1.30 m, 2.21 m	28.7	2.04 m, 2.18 dd (6.50, 8.00)	27.8	1.98 m; 1.58 m	24.7
7	3.14 brd (5.00)	73.5	3.38 brd (10.50)	78.3	3.09 dd (7.5, 2.5)	84.0
8	—	78.5	—	74.5	—	70.0
9	1.43 m; 2.00 m	37.0	1.51 m; 1.79 m	43.1	1.90 m, 1.59 m	40.4
10	1.51 m; 1.85 m	28.2	1.47 m; 1.85 m	28.9	1.58 m, 1.51 m	23.7
11	3.57 brd (10.00)	76.2	3.16 d (7.50)	80.0	3.29 brd (10.00)	80.2
12	—	78.0	—	80.1	—	73.1
13	1.62 m; 1.78 m	31.0	1.49 m, 1.96 m	34.7	2.35 m, 2.24 m	36.3
14	2.43 brt (12.20), 2.57 m	20.8	1.99 m, 2.41 m	20.8	2.05 m; 2.53 m	20.3
15	—	121.8	—	122.3	—	123.1
16	—	174.0	—	176.0	—	175.5
17	1.83 s	7.8	1.83 s	8.8	1.85 s	8.9
18	2.02 s	20.8	1.91 brs	17.1	1.83 brs	16.7
19	1.11 s	13.6	1.39 s	20.7	1.17 s	20.3
20	1.03 s	17.0	1.03 s	17.6	1.16 s	23.3
21	3.20 s	48.3				

a *J* values (Hz) in parentheses, obtained at 500 and 125 MHz for ^1^H and ^13^C NMR, respectively.

b Recorded in CDCl_3_.

c Recorded in CDCl_3_–CD_3_OD (9 : 1).

The signal at *δ*_H_ 5.45 (d; *J* = 10.0 Hz) correlated with a proton signal at *δ*_H_ 5.14 (d, *J* = 10.0 Hz) and quaternary olefinic carbons at *δ*_C_ 147.0 and *δ*_C_ 163.0 in DQF-COSY and HMBC (Fig. 2), respectively, allowed for the assignments of H-2, H-3, C-4 and C-1, respectively.^8–10,23–25^ Correlations in the HMBC spectrum showed several informative connections: H-3 to carbon signals at *δ*_C_ 13.6 (q, olefinic) *δ*_C_ 34.6 (t), allowed for the assignment of H-18 (*δ*_H_ 2.02, s) and H-5 (*δ*_H_ 2.37, brd, *J* = 14.0), respectively; methyl signal *δ*_H_ 1.83 (s) to C-1 and carbon signal at *δ*_C_ 174.0 (C=O) attributed to H-17 and C-16, respectively as well as supporting the location of C-1/C-2 lactone ring; methyl singlet at *δ*_H_ 1.11 to *δ*_C_ 73.5 (C-7), *δ*_C_ 37.0 and 78.5 allowed for the location of H_3_-19 (*δ*_C_ 13.6), C-9 and C-8, respectively; the oxygenated broad doublet at *δ*_H_ 3.57 (*δ*_C_ 79.0) to C-9 and C-20, was assigned to H-11. The assignment of H-7, H_2_-6 and C-5 was detected through the correlation of the oxygenated methine signal at *δ*_H_ 3.14 (brd, *J* = 5.0) to a methylene multiplet at *δ*_H_ 1.30/2.21 and a carbon signal at *δ*_C_ 34.6 in DQF-COSY and HMBC, respectively. Additionally, a correlation was detected in DQF-COSY between H-13 (*δ*_H_ 1.78, m) and H-14 (*δ*_H_ 2.43, brt, *J* = 12.2) as well as to C-20 in HMBC analyses (Fig. 2). An HMBC correlation established the site of a methoxy group (*δ*_H_ 3.20 s, *δ*_C_ 48.3 q) at C-8.

The planar structure assignment of 1 and the C-7/C-12 ether linkage were proposed by 1D, 2D NMR and HREIMS data. The data comparison with those of sarcoehrenbergilid A, as previously reported,^23^ suggested that 1 and sarcoehrenbergilid A,^23^ differ only in stereochemistry.

**Fig. 3:**
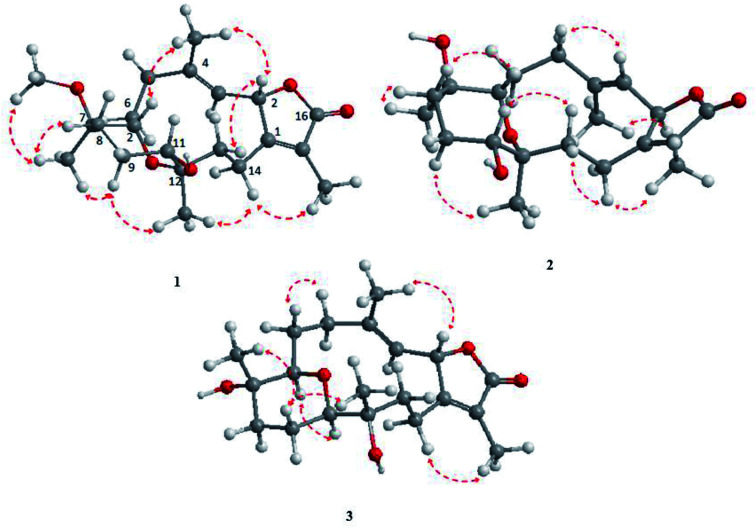
Selected NOESY correlations for 1–3.

**Fig. 4:**
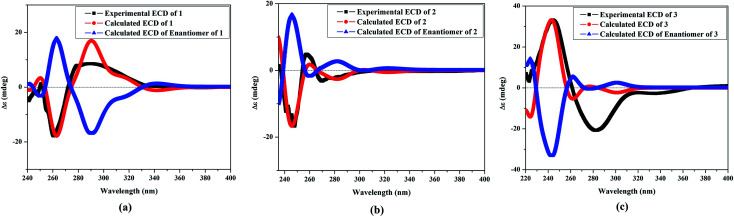
Experimental electronic circular dichroism (ECD in MeOH): (a) 1 compared with the TDDFT-simulated ECD spectra of 2*S*,7S,12*S*-diepoxy-11*R*-hydroxy-8*R*-methoxy-16-keto-cembra-1*Z*,3*E*-diene and 2*R*,7*R*,12*R*-diepoxy-11*S*-hydroxy-8*S*-methoxy-16-keto-cembra-1*E*,3*Z*-diene; (b) 2 compared with the TDDFT-simulated ECD spectra of 2*R*,7*S*,12*S*-diepoxy-11*R*-hydroxy-8*R*-methoxy-16-keto-cembra-1*Z*,3*E*-diene and 2*S*,7*R*,12*R*-diepoxy-11*S*-hydroxy-8*S*-methoxy-16-keto-cembra-1*E*,3*Z*-diene; and (c) 3 compared with the TDDFT-simulated ECD spectra of 2*S*,7*R*,11*R*-diepoxy-12*S*-hydroxy-8*S*-methoxy-16-keto-cembra-1*Z*,3*E*-diene and 2*R*,7*S*,11*S*-diepoxy-12*R*-hydroxy-8*R*-methoxy-16-keto-cembra-1*E*,3*Z*-diene.

The NOESY spectrum revealed that a γ-lactone at H-2 (*δ*_H_ 5.45, d, *J* = 10.0 Hz) correlated with CH_3_-18 (*δ*_H_ 2.02, s); a vicinal coupling with H-3 established a trans configuration and a β-orientation for H-2.^8^ NOSEY correlations were observed between three methyl groups with alpha protons (*e.g.*, CH_3_-20 with H-10a, CH_3_-19 with H-6a/H-10a, and CH_3_-17 with H-14a) (Fig. 3). H-7 and H-11 was assigned to a β-configuration based on NOSEY correlations with H-5b and H-14b, respectively. Absolute configuration was established by experimental and TDDFT-simulated ECD spectra. All possible conformations of 1 within energy window of 10 kcal mol^−1^ were generated and optimized at B3LYP/6-31G* level of theory. The first 50 excitation states were then computed based on time-dependent density-functional theory (TDDFT) at B3LYP/6-31G* level in methanol by the PCM model. The generated TDDFT-ECD spectra were Boltzmann-weighted and compared to the experimental spectrum (Fig. 4). The TDDFT-simulated ECD spectrum was in a good agreement with the corresponding experimental ECD spectra (Fig. 4). This comparison revealed the absolute configuration and therefore 1 was assigned as 2*S*,16:7*S*,12*S*-diepoxy-11*R*-hydroxy-8*R*-methoxy-16-keto-cembra-1*Z*,3*E*-diene (sarcoehrenbergilid D).

Compound 2 was isolated as a white powder with a negative optical rotation of [*α*]^25^_D_ = −5.4 (*c* 0.03, CHCl_3_). The molecular formula (C_20_H_30_O_5_) was detected by high resolution electron ionization (HREIMS) spectrum (*m*/*z* 350.2094 [M]^+^, calcd 350.2093). HREIMS analysis exhibited a molecular ion peak at *m*/*z* 350.2094 [M]^+^ (calcd) The IR spectrum showed characteristic bands at *ν*_max_ 3445 cm^−1^ and 1747 cm^−1^ for hydroxyl and keto groups, respectively. The ^13^C NMR spectrum revealed twenty carbon signals (Table 1) classified by DEPT as six quaternary, four methines, six methylenes and four methyls carbons. 1D and 2D NMR spectroscopic data were quite close to sarcoehrenbergilid A,^23^ a formerly isolated diterpenoid from *S. ehrenbergi* except for an absence of methoxyl groups. For 2 there is an upfield carbon signal at *δ*_C_ 74.5 and a downfield methyl signal at *δ*_C_ 20.7 for C-8 and CH_3_-19, respectively.

Stereochemistry was established based on coupling constants and NOESY experiments (Fig. 3). NOESY correlation indicated that 2 has the same relative stereochemistry as sarcoehrenbergilid A.^23^ To determine absolute configuration, TDDFT-ECD calculations were performed on the 2*R*,7*S*,8*R*,11*R*,12*S*- and 2*S*,7*R*,8*S*,11*S*,12*R*-enantiomers. The final Boltzmann-weighted TDDFT-ECD spectra were then compared to the corresponding experimental ECD curve (Fig. 4). According to the data depicted in Fig. 4, the 2*R*,7*S*,8*R*,11*R*,12*S*-enantiomer reproduced all the transitions of the experimental ECD spectrum. Therefore, 2 was assigned as 2*R*,16:7*S*,12*S*-diepoxy-11*R*-hydroxy-8*R*-methoxy-16-keto-cembra-1*Z*,3*E*-diene (sarcoehrenbergilid E). Compound 3 was isolated as a colorless oil with a negative optical rotation of [*α*]^25^_D_ = −10.8 (*c* 0.01, CHCl_3_). The molecular formula of C_20_H_30_O_5_ was detected by high resolution electron ionization (HREIMS) analysis (*m*/*z* 332.1993 [M − H_2_O^+^], calcd 332.1998).

The IR spectrum showed characteristic bands at *ν*_max_ 3445 cm^−1^ and 1747 cm^−1^ for hydroxyl and keto groups, respectively. The ^13^C NMR spectrum (Table 1) showed 20 carbon resonances classified by DEPT analysis as four methyls, six methylenes, four methines and six quaternary carbons. The 1D (^1^H, ^13^C) as well as 2D NMR (^1^H–^1^H COSY, HSQC, and HMBC) (Fig. 2) spectroscopic data closely matches a previously reported cemberene compound.^26^ The NOESY correlation (Fig. 3) as well as the ^1^H and ^13^C NMR analyses indicated that 3 is a C-2 epimer of the previously isolated sarcophyolide E^26^ through the clear difference in downfield shift of H-3 (*δ*_H_ 5.10, d, *J* = 10.0). Additionally, several carbon signals showed downfield chemical shift in comparison of sarcophyolide E: *δ*_C_ 37.0/36.2 (C-5), 73.1/71.8 (C-12), 123.1/121.7 (C-15), and 175.5/174.9 (C-17), respectively. The carbon signals at *δ*_C_ 163.9 (C-1) and 36.3 (C-13) showed upfield chemical shift in comparison with sarcophyolide E [*δ*_C_ 165.8 (C-1) and 37.3 (C-13)].

The relative configuration for 3 was established based on coupling constants and NOESY experiments (Fig. 3). A NOE correlation between H-7 (*δ*_H_ 3.09 dd, *J* = 7.5, 2.5) and H-11 (*δ*_H_ 3.29 brd, *J* = 10.0) established an alpha linkage for the ether bridge between C-7 and C-11. The NOE correlations between H-3 and the γ-lactone-(H-2) as well as vicinal coupling constant indicated a *trans*-geometry for H-2 and H-3 of the olefinic bond (Fig. 3). As expected, the experimental ECD for 3 and published compound, sarcophyolide E,^26^ showed inverted direction for positive and negative cotton effect as well as optical rotation (Fig. 4). These data indicated that 3 is the C-2 epimer of sarcophyolides E. Thus, 3 was confirmed to be 2*S*,16:7*R*,11*R*-diepoxy-12*S*-hydroxy-8*S*-methoxy-16-keto-cembra-1*Z*,3*E*-diene (sarcoehrenbergilid F).

**Fig. 5:**
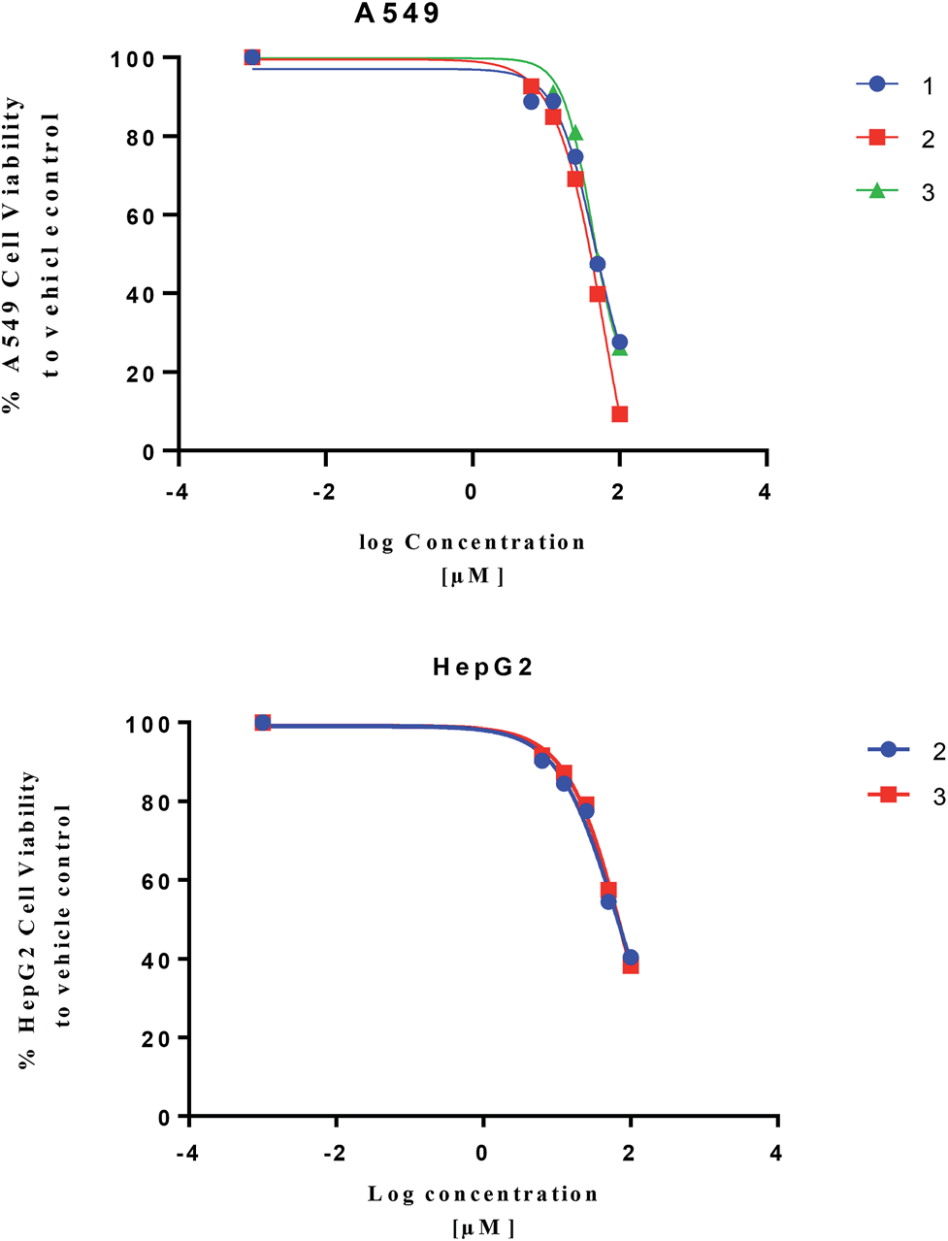
Cytotoxicity assay of 1–3 based on MTT-reduction assay.

Isolated metabolites 1–3 were tested for cytotoxic activity toward lung (A549), colon (Caco-2) and liver (HepG2) human cancer cell lines based on an MTT reduction assay (Fig. 5). Compounds 1–3 showed most potent activity toward A549 cells with IC_25_ values of 23.3, 27.3, and 25.4 μM, respectively. Compound 2 and 3 showed weaker activity toward liver (HepG2) human cancer cell lines with IC_25_ values of 22.6 and 31.8 μM, respectively. The treated human colon cancer cells (Caco-2) cell viability was over 100% for all the isolated compounds (IC_25_ > 100 μM). Since primary necrosis is not easily differentiated from secondary necrosis that occurs with apoptosis,^27^ the mode of action will not be considered. To differentiate these distinct biological events requires apoptotic assays accompanying necrosis measurements. A combined necrosis/apoptotic time-course will be presented in a subsequent study to elaborate on mode of action.

## 3. Experimental section

### 3.1. General experimental procedures

Circular dichroism was measured on JASCO 810 spectropolarimeter. HREIMS data were collected on a JEOL JMS-700 instrument (Tokyo, Japan). NMR spectra were recorded on a Bruker 500 NMR spectrometer (Japan). JASCO P-2200 polarimeter and JASCO FT/IR-6300 spectrometer was used for optical rotation and infrared measurements, respectively.

Normal-phase silica gel 60 (230–400) column chromatography (CC) as well as aluminum TLC plates (silica gel 60 F_254_) (Merck, Darmstadt, Germany) were used for purification and monitoring spotting, respectively. A H_2_SO_4_ : MeOH (1 : 9) spraying reagent was used for spot visualization after heating. HPLC purification was performed using Shimadzu HPLC-RID-10A with YMC-Pack ODS-A analytical (250 × 4.6 mm i.d.) and preparative (250 × 20 mm i.d.) columns (YMC, Tokyo, Japan) for separation.

### 3.2. Animal material

*Sarcophyton ehrenbergi* coral was collected from the Red Sea on the Egyptian coast at Hurghada, in March 2016 and identified by Dr M. Al-Hammady. A voucher specimen (03RS27/1) was deposited in the National Institute of Oceanography and Fisheries, marine biological station, Hurghada, Egypt.

### 3.3. Extraction and isolation

Sliced frozen soft coral (2 kg, total wet weight) were extracted with CH_2_Cl_2_ : MeOH (1 : 1, v/v) at room temperature (3 L × 4 times). Isolation protocol was performed as described previously by Hegazy *et al.*, 2017 (ref. 23) to afford 1 (5.5 mg), 2 (4 mg) and 3 (6 mg).

#### 3.3.1 Sarcoehrenbergilid D (1)

White powder; [*α*]^25^_D_ +10.8 (*c* 0.02, CHCl_3_); FT-IR (KBr) *ν*_max_: 3435, 2941, 1748, 1462, and 1224 cm^−1^; ^1^H and ^13^C NMR data, see Table 1; HREIMS *m*/*z* 346.2127 [M − H_2_O]^+^ (calcd 346.2149).

#### 3.3.2 Sarcoehrenbergilid E (2)

White powder; [*α*]^25^_D_ −5.4 (*c* 0.03, CHCl_3_); FT-IR (KBr) *ν*_max_: 3433, 2938, 1743, 1446, and 1218 cm^−1^; ^1^H and ^13^C NMR data, see Table 1; HREIMS *m*/*z* 350.2094 [M]^+^ (calcd 350.2093).

#### 3.3.3 Sarcoehrenbergilid F (3)

White amorphous powder; [*α*]^25^_D_ −10.8 (*c* 0.01, CHCl_3_); FT-IR (KBr) *ν*_max_: 3441, 2932, 1742, 1448, and 1229 cm^−1^; ^1^H and ^13^C NMR data, see Table 1; HREIMS *m*/*z* 332.1993 [(M − H_2_O)^+^] (calcd 332.1998).

### 3.4. Biological activity

#### 3.4.1 Cell lines

Three human cancer cell lines, A549 (non-small cell lung adenocarcinoma), Caco-2 (colon adenocarcinoma) and HepG2 (hepatocellular carcinoma) (ATCC®) were assayed with the purified compounds. All cell lines were cultured in Dulbecco's modified Eagle's medium (DMEM) supplemented with 10% (FBS fetal bovine serum), 1% penicillin and incubated in 5% CO_2_ at 37 °C.

#### 3.4.2 MTT cytotoxicity assay

The cytotoxicity of tested compounds was investigated by a MTT assay. Cell lines were seeded and incubated overnight allowing cell adhesion to the plate well (5 × 10^3^ cells per well; 96-well plate in a volume of 100 μL). To generate concentration-dependent curves, sample concentration was varied (100, 50, 25, 12.5, 6.25 μM) for a total well volume of 200 μL for 48 h. MTT solution (5 mg ml^−1^) was added (100 μL per well) for 90 min before measurements.^28,29^ After medium removal, dark blue formazan crystals formed in viable cells were dissolved in 100 μL of DMSO, followed by shaking for 10 min (200 rpm). The absorbance was recorded at 492 nm using a microplate reader (Sunrise™ microplate reader, Tecan Austria Gmbh, Grödig, Austria) for cell viability measurement. IC_25_ values were expressed as a concentration of tested compound that inhibits 50% cell growth in comparison with a vehicle control (quadrate to octuplet treatment) by non-linear regression model analyses using GraphPad Prism® v 6.0 software.

### 3.5. Computational functional theory calculations

Conformational analysis was performed using Omega2 software^30^ to obtain the possible conformers for 1–3 within energy window value of 10 kcal mol^−1^. All resulting conformers were optimized at B3LYP/6-31G* level of theory using Gaussian09 software.^30^ Frequency calculations were then performed on the optimized structures to ensure the nature of the local minima as well as to estimate the Gibbs free energies. Time-dependent density functional theory (TDDFT) calculations with incorporating a polarizable continuum model (PCM) using methanol as a solvent were carried out at the B3LYP/6-31G* level of theory to calculate the first fifty excitation states. Electronic circular dichroism (ECD) spectra were finally generated using SpecDis 1.71 (SpecDis 2017 (ref. 31 and 32)) by applying Gaussian band shapes with sigma = 0.20–30 eV. The generated ECD spectra were Boltzmann-averaged.

## 4. Conclusions

Cembrene diterpenoids (1–3) were isolated and identified from the *S. ehrenbergi* soft coral. The isolated compounds were tested against three human cancer cell lines, which resulted in 2 being the most potent compound against lung A549 cancer cell. The absolute stereochemistry of 1–3 were confirmed by comparing experimental and TDDFT-simulated ECD spectra.

## Conflicts of interest

There are no conflicts to declare.

## Supplementary Material

RA-009-C9RA04158C-s001
