# Supplementary material for: Sarcoehrenbergilides D–F: cytotoxic cembrene diterpenoids from the soft coral Sarcophyton ehrenbergi
Source: RSC Adv. 2019 Aug 29;9(47):27183–9. doi: 10.1039/c9ra04158c (PMC9070672; doi:10.1039/c9ra04158c)
Supplement: RA-009-C9RA04158C-s001 [file RA-009-C9RA04158C-s001.pdf]

Electronic Supplementary Material (ESI) for RSC Advances.
This journal is © The Royal Society of Chemistry 2019

Supplementary Materials

Sarcoehrenbergilides D-F: Cytotoxic Cembrene Diterpenoids from the Soft Coral *Sarcophyton ehrenbergi*

Mohamed-Elamir F. Hegazy[1,2], Tarik A. Mohamed[2], Abdelsamed I. Elshamy[3,4], Ahmed R. Hamed[2], Mahmoud A. A. Ibrahim[5], Shinji Ohta[6], Akemi Umeyama[4], Paul W. Paré[7*], and Thomas Efferth[1*]

[1] Department of Pharmaceutical Biology, Institute of Pharmacy and Biochemistry, Johannes Gutenberg University, Staudinger Weg 5, 55128 Mainz, Germany
[2] Chemistry of Medicinal Plants Department, National Research Centre, El-Tahrir St., Dokki, Giza 12622, Egypt; E-Mail: elamir77@live.com; tarik.nrc83@yahoo.com; n1ragab2004@yahoo.com
[3] Natural Compound Chemistry Department, National Research Centre, El-Tahrir St., Dokki, Giza 12622, Egypt; E-Mail: elshamynrc@yahoo.com
[4] Faculty of Pharmaceutical Sciences, Tokushima Bunri University, Yamashiro-cho, Tokushima, 770-8514, Japan E-Mail: umeyama@ph.bunri-u.ac.jp
[5] Computational Chemistry Laboratory, Chemistry Department, Faculty of Science, Minia University, Minia 61519, Egypt; E-Mail: m.ibrahim@compchem.net
[6] Graduate School of Biosphere Science, Hiroshima University, 1-7-1 Kagamiyama, Higashi-Hiroshima 739-8521, Japan; E-Mail: ohta@hiroshima-u.ac.jp
[7] Department of Chemistry and Biochemistry, Texas Tech University, Lubbock, TX 79409, USA

Supporting data Page

S1: FTIR of **1** 3
S2: LREIMS of **1** 4
S3: HREIMS of **1**.. 5
S4: ^{1}H NMR of **1**.. 6
S5: ^{13}C NMR of **1** 7
S6: DEPT-135 of **1**.. 8
S7: HSQC of **1**.. 9
S8: HMBC of **1**.. 10
S9: ^{1}H ^{1}H COSY of **1**.. 11
S10: NOESY of **1**.. 12
S11: FTIR of **2** 13
S12: LREIMS of **2** 14
S13: HREIMS of **2**.. 15
S14: ^{1}H NMR of **2**.. 16
S15: ^{13}C NMR of **2** 17
S16: DEPT-135 of **2**.. 18

S17: HSQC of **2**………………………………………………………………. 19
S18: HMBC of **2**……………………………………………………………… 20
S19: ^{1}H ^{1}H COSY of **2**…………………………………………………… 21
S20: NOESY of **2**…………………………………………………………….. 22
S21: FT-IR of **3** ……………………………………………………………. 23
S22: LREIMS of **3** …………………………………………………………….. 24
S23: HREIMS of **3**…………………………………………………………….. 25
S24: ^{1}H NMR of **3**…………………………………………………………….. 26
S25: ^{13}C NMR of **3** ………………………………………………………… 27
S26: DEPT-135 of **3**……………………………………………………….. 28
S27: HSQC of **3**………………………………………………………………. 29
S28: HMBC of **3**…………………..……………………………..……. 30
S29: ^{1}H ^{1}H COSY of **3**…………………………………………………… 31
S30: NOESY of **3**…………………………………………………………. 32

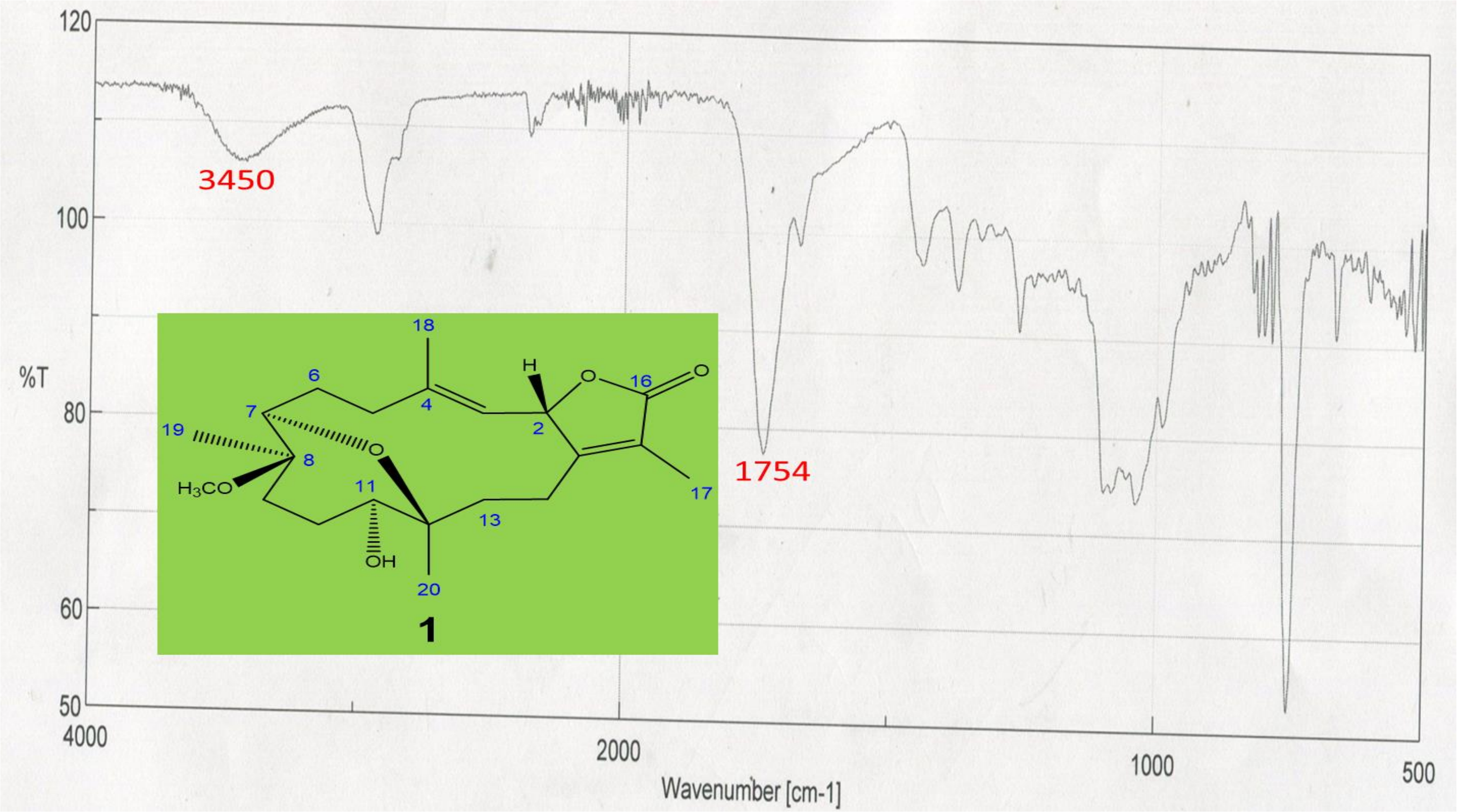
120
100
80
60
50
%T
4000
2000
1000
500
Wavenumber [cm-1]
3450
1754
18
6
4
H
O
16
O
7
2
19
8
H_3CO
11
13
17
OH
20
1

S1: FT-IR of **1**

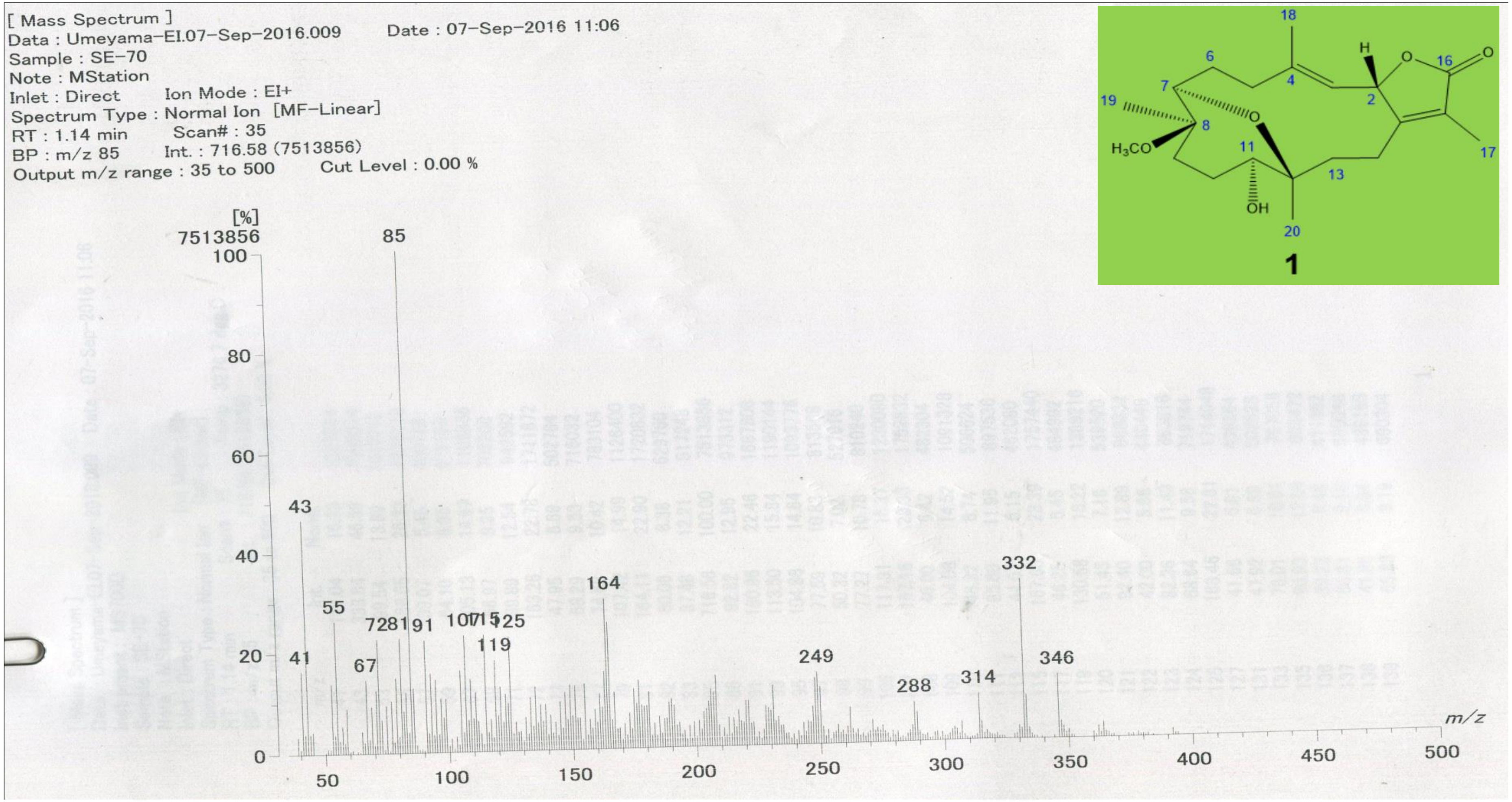

S2: LREIMS of **1**

Data : Umeyama-EIHR.07-Sep-2016.005   Date : 07-Sep-2016 15:18

Instrument : MS700D

Sample : SE-70

Note : MStation

Inlet : Direct   Ion Mode : EI+

RT : 1.24 min   Scan# : 32

Elements : C 150/0, H 250/0, O 50/0

Mass Tolerance : 5mmu

Unsaturation (U.S.) : 0.0 - 15.0

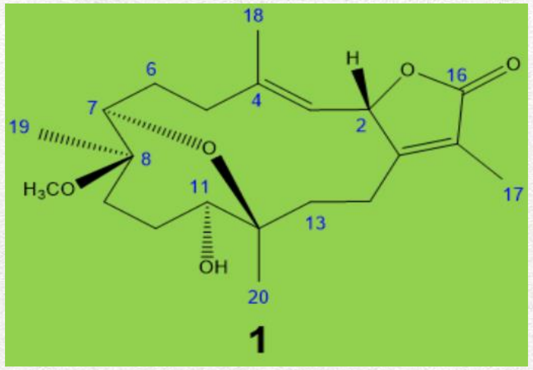

1

	Observed m/z	Int%	Err[ppm / mmu]	U.S.	Composition
1	332.2002	17.70	+4.3 / +1.4	7.0	C20 H28 O4
2	346.2127	8.01	-4.9 / -1.7	7.0	C21 H30 O4

S3: HREIMS of **1**

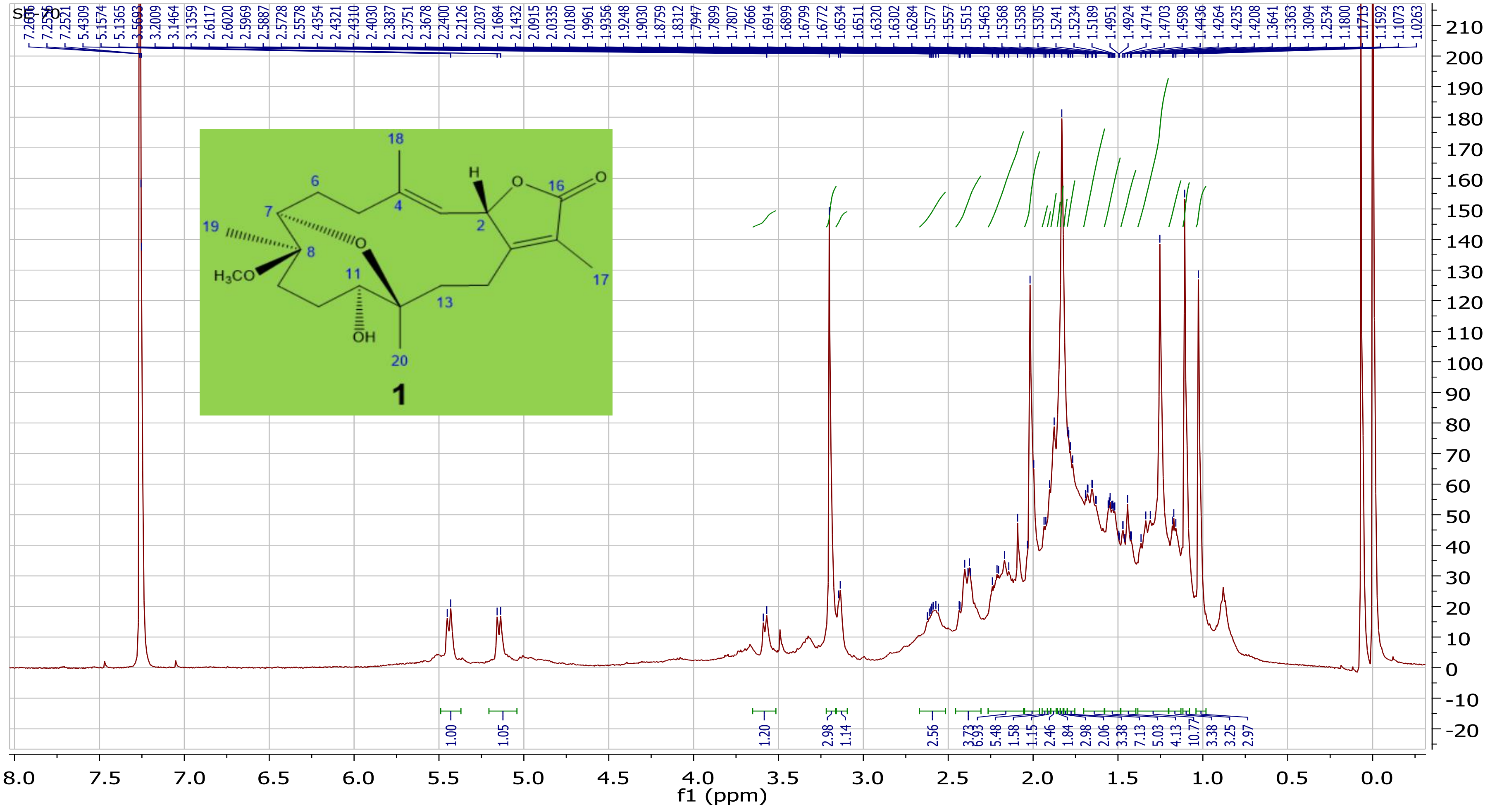

S4: ^{1}H NMR of **1**

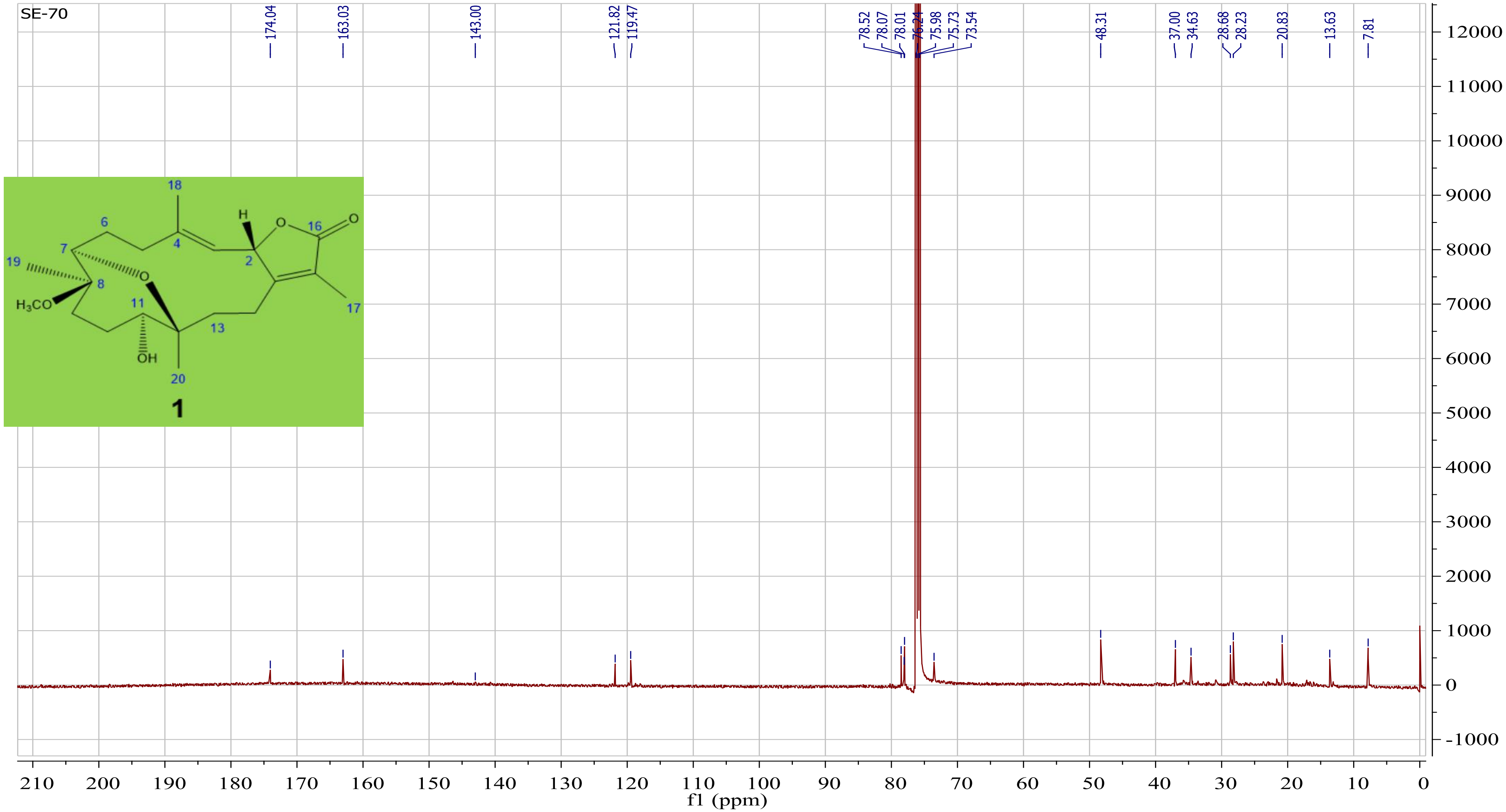

S5: ^{13}C NMR of **1**

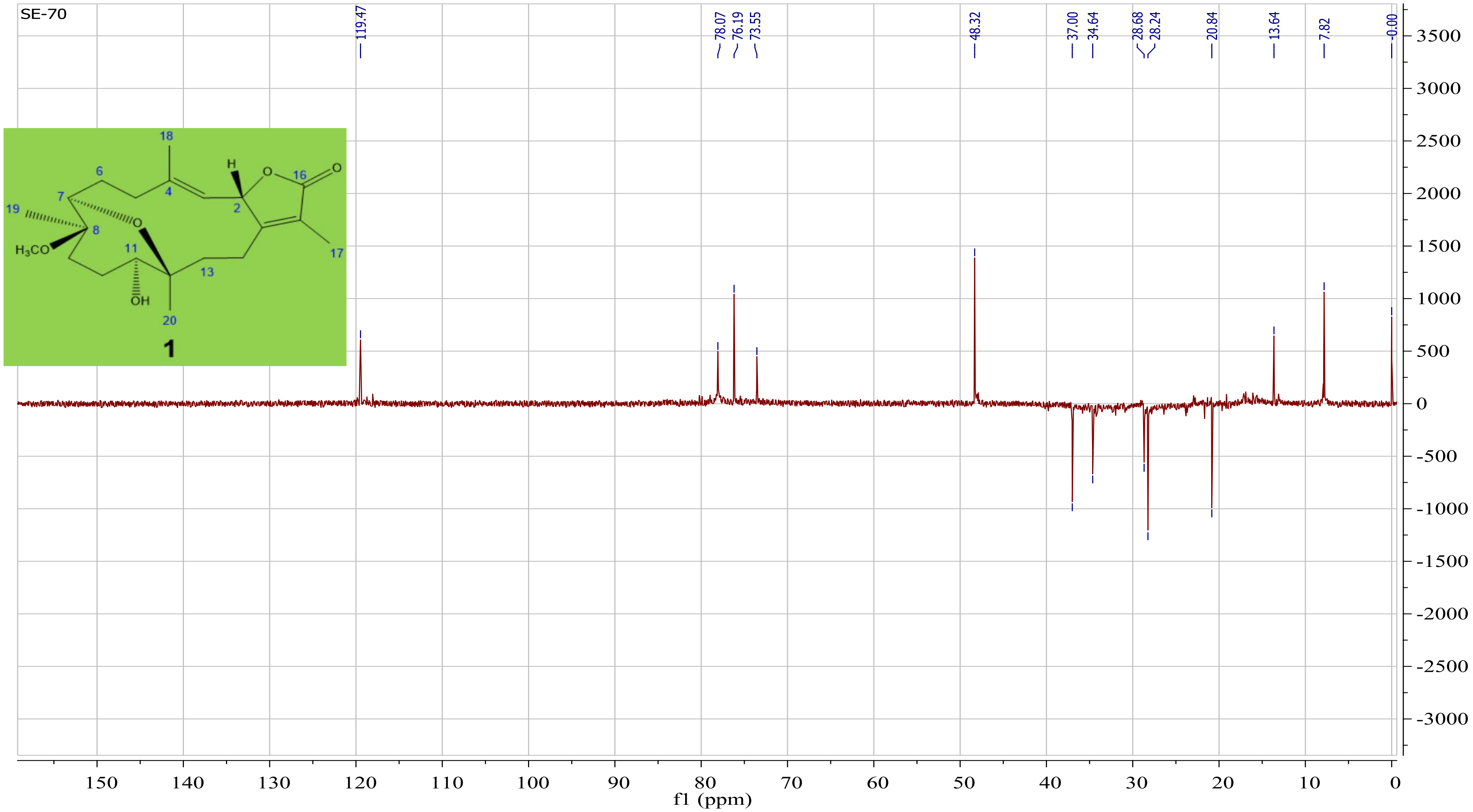

S6: DEPT-135 of **1**

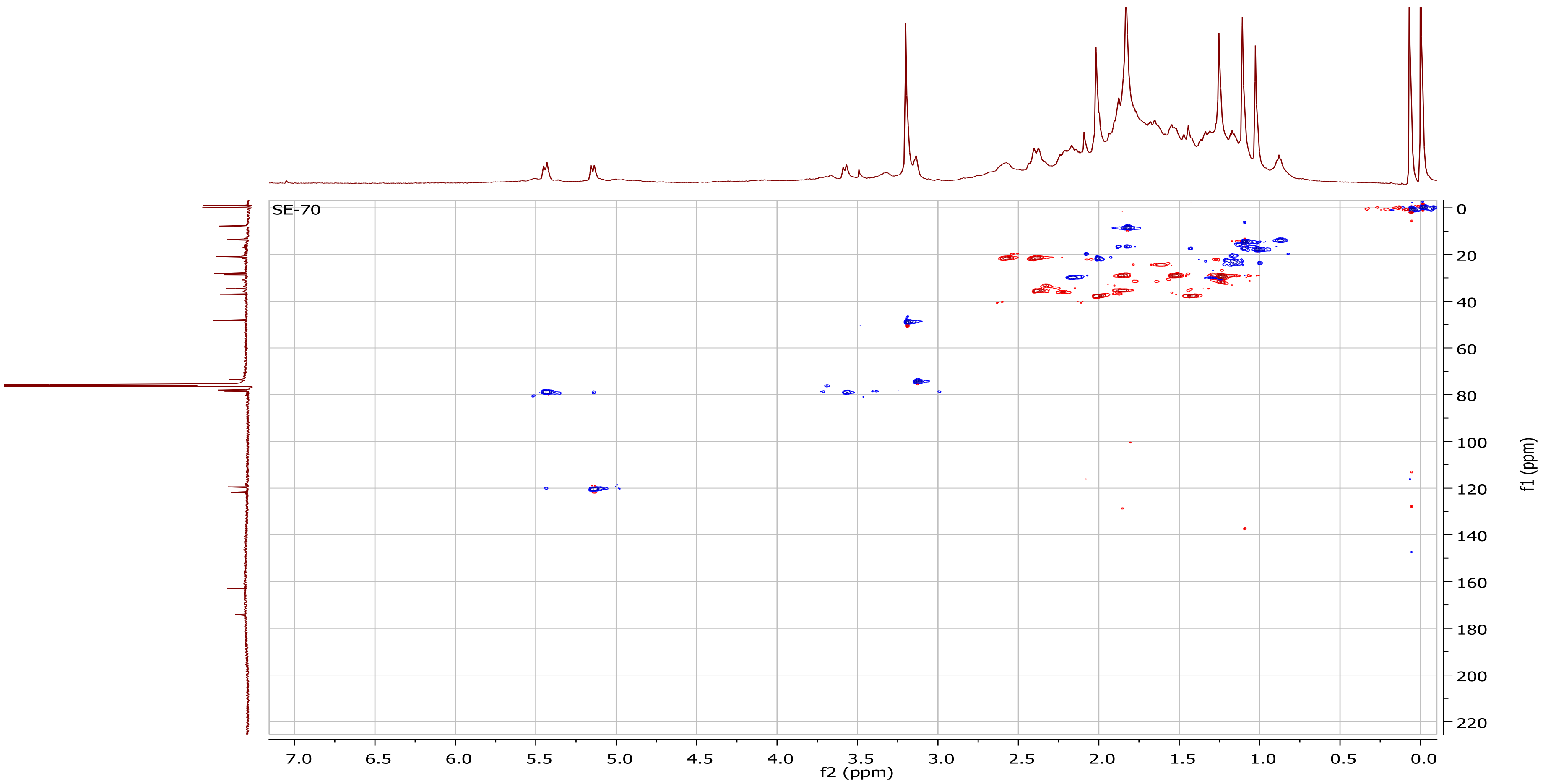
SE-70
f2 (ppm)
f1 (ppm)
7.0
6.5
6.0
5.5
5.0
4.5
4.0
3.5
3.0
2.5
2.0
1.5
1.0
0.5
0.0
0
20
40
60
80
100
120
140
160
180
200
220

S7: HSQC of **1**

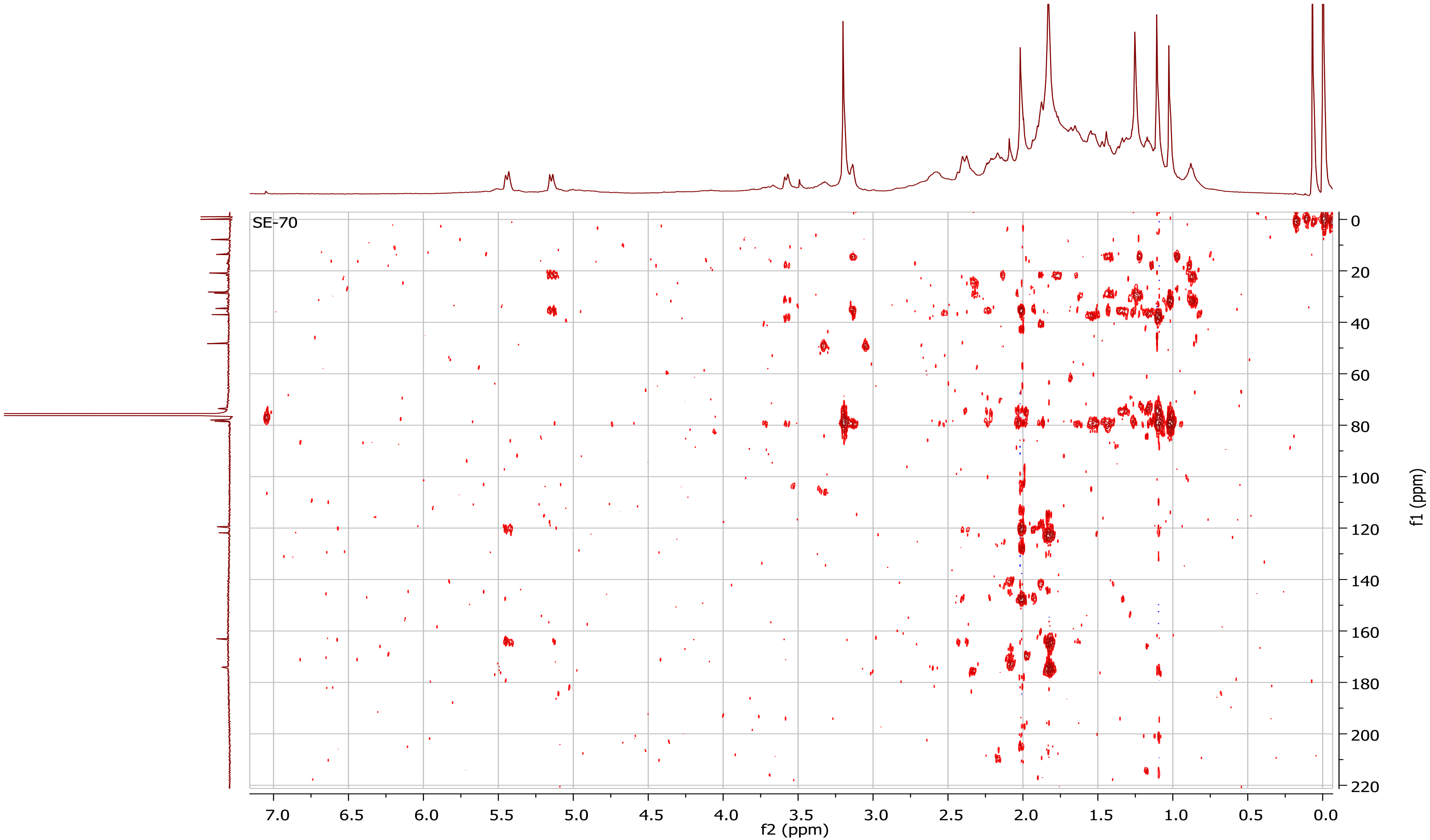

SE-70
7.0
6.5
6.0
5.5
5.0
4.5
4.0
3.5
3.0
2.5
2.0
1.5
1.0
0.5
0.0
f2 (ppm)
0
20
40
60
80
100
120
140
160
180
200
220
f1 (ppm)

S8: HMBC of **1**

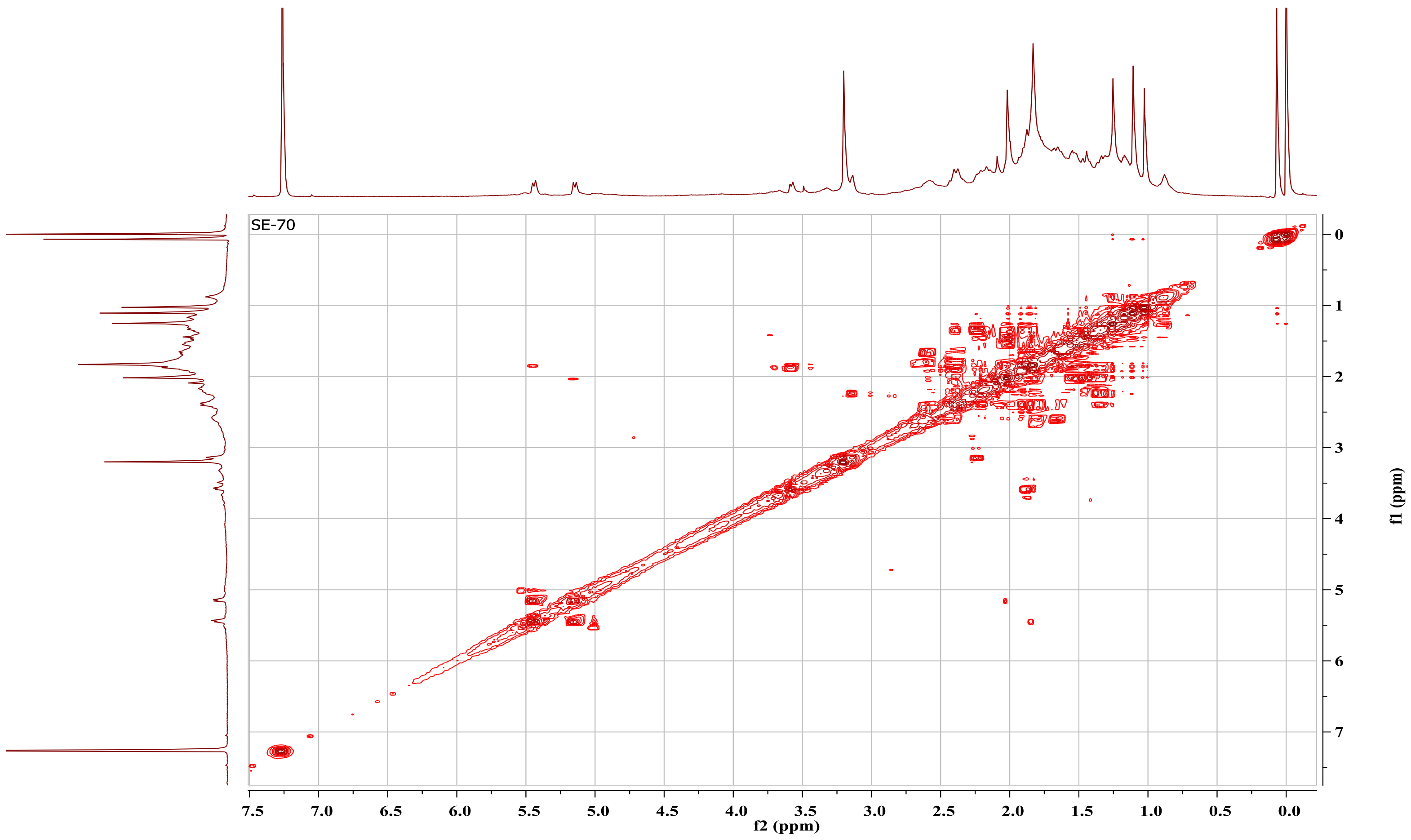

S9: ^{1}H ^{1}H COSY of **1**

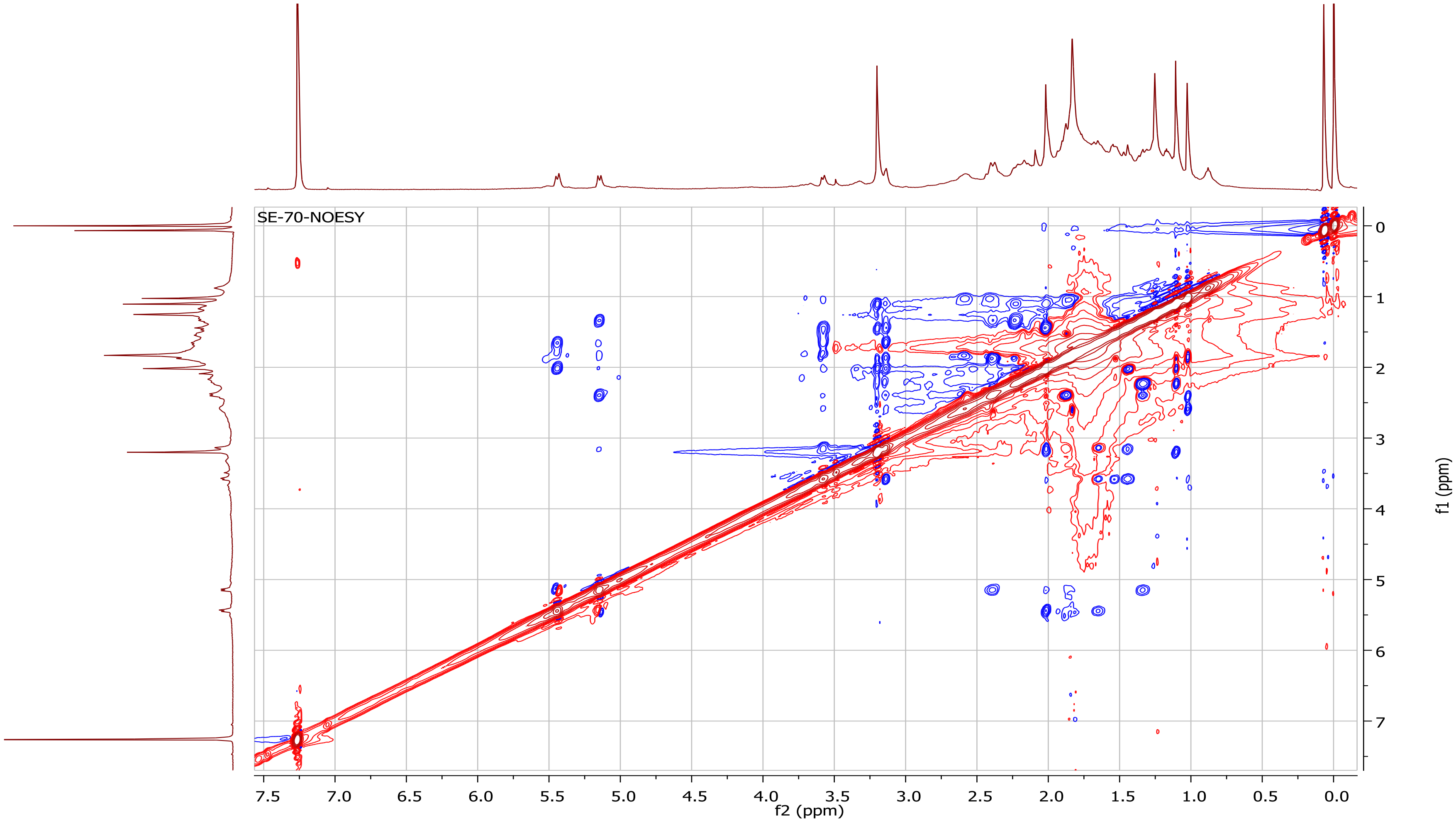

S10: NOESY of **1**

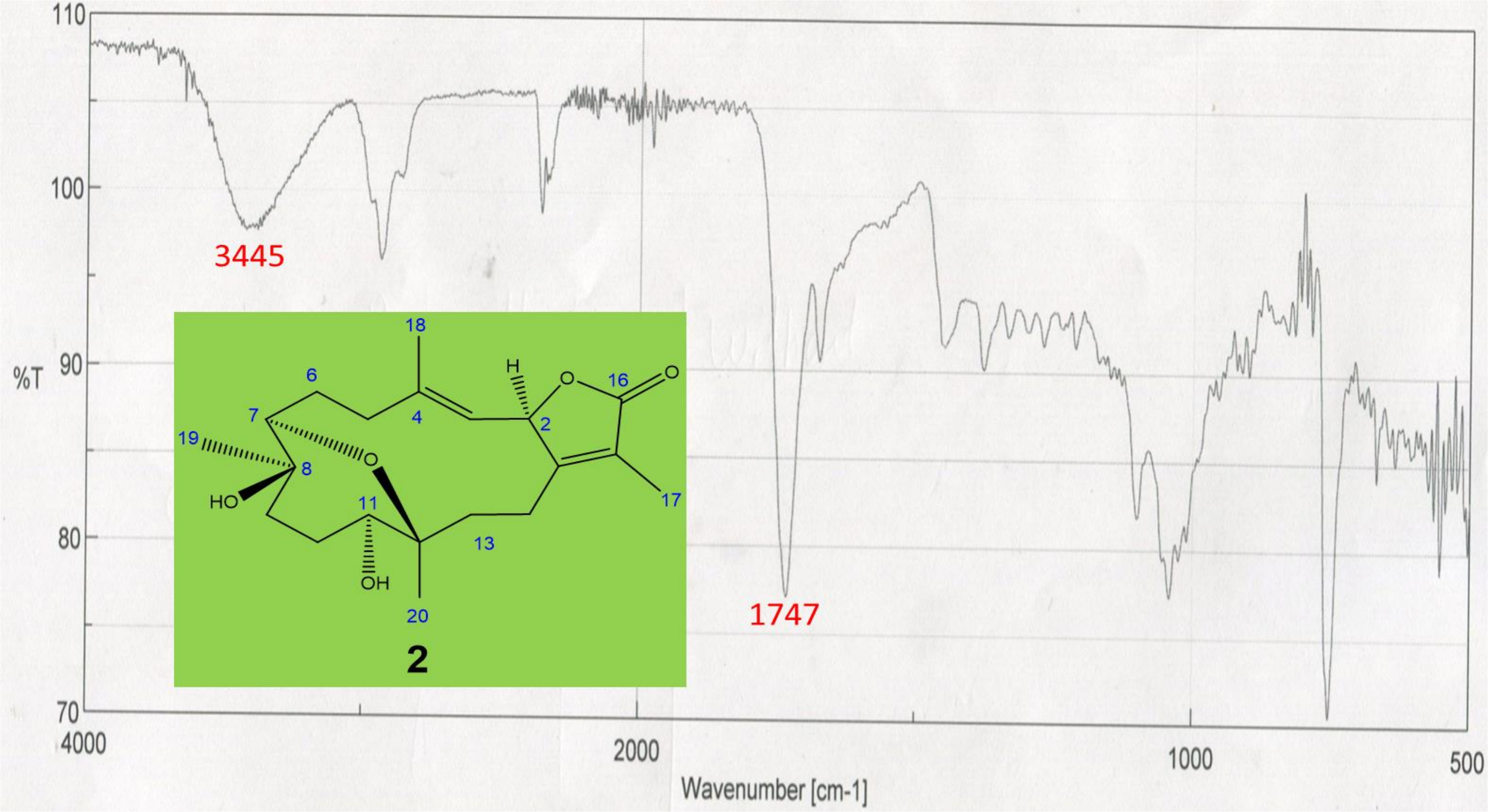
110
100
90
80
70
%T
4000
2000
1000
500
Wavenumber [cm-1]
3445
1747
18
6
H
O
16
O
7
4
2
19
O
8
HO
11
17
13
OH
20
2

S11: FT-IR of **2**

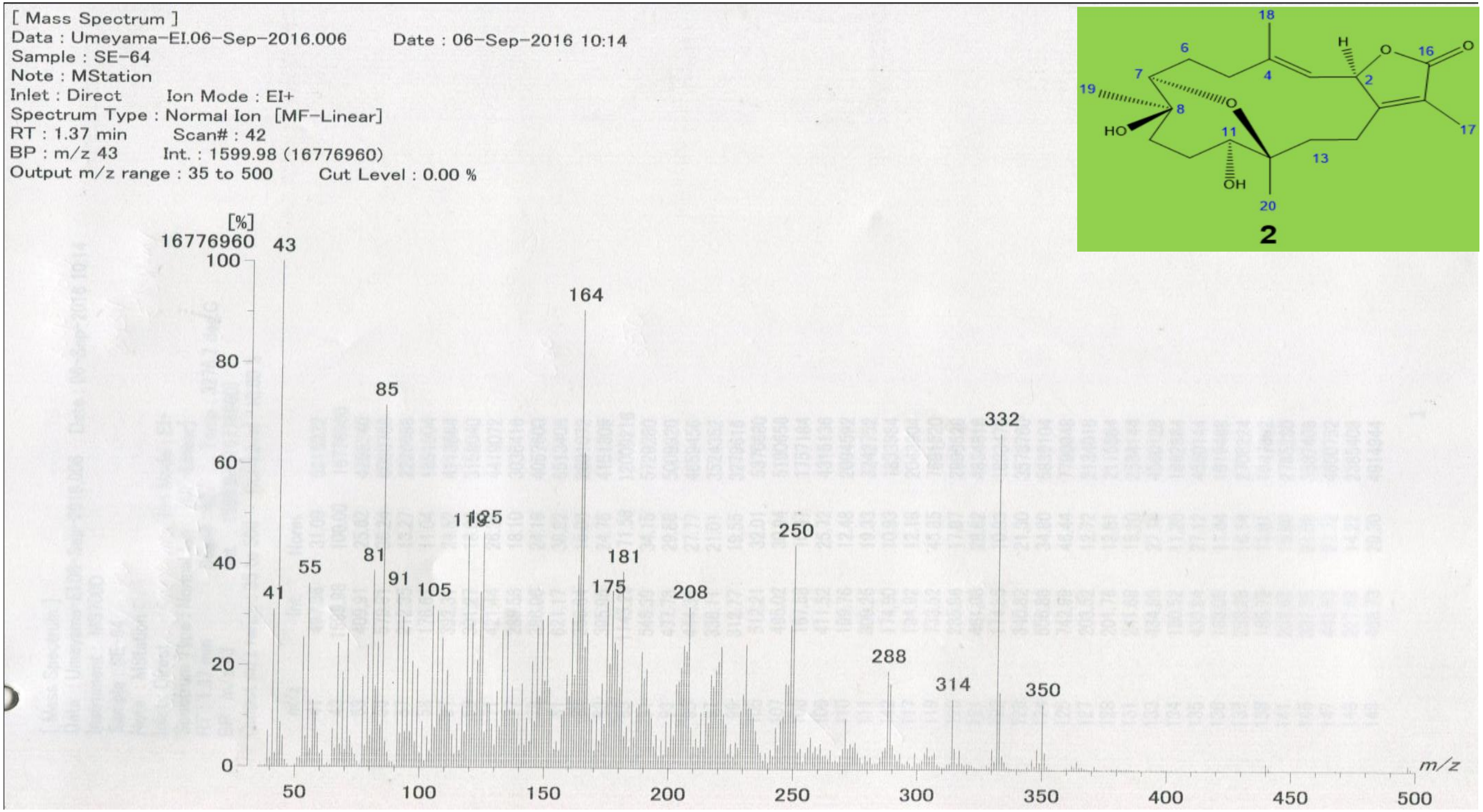

S12: LREIMS of **2**

Data : Umeyama–EIHR.07–Sep–2016.003   Date : 07–Sep–2016 09:09

Instrument : MS700D

Sample : SE–64

Note : MStation

Inlet : Direct   Ion Mode : EI+

RT : 1.88 min   Scan# : 48

Elements : C 150/0, H 250/0, O 50/0

Mass Tolerance : 5mmu

Unsaturation (U.S.) : 0.0 – 15.0

	Observed m/z	Int%	Err[ppm / mmu]	U.S.	Composition
1	332.2008	100.00	+6.1 / +2.0	7.0	C20 H28 O4
2	350.2094	22.70	+0.2 / +0.1	6.0	C20 H30 O5

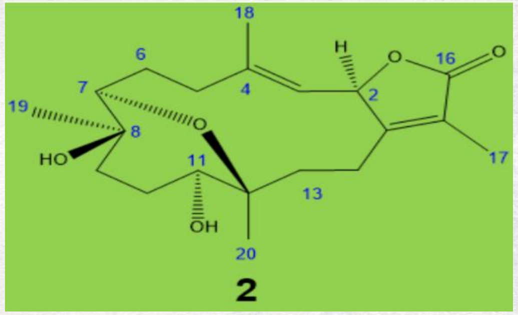

2

S13: HREIMS of **2**

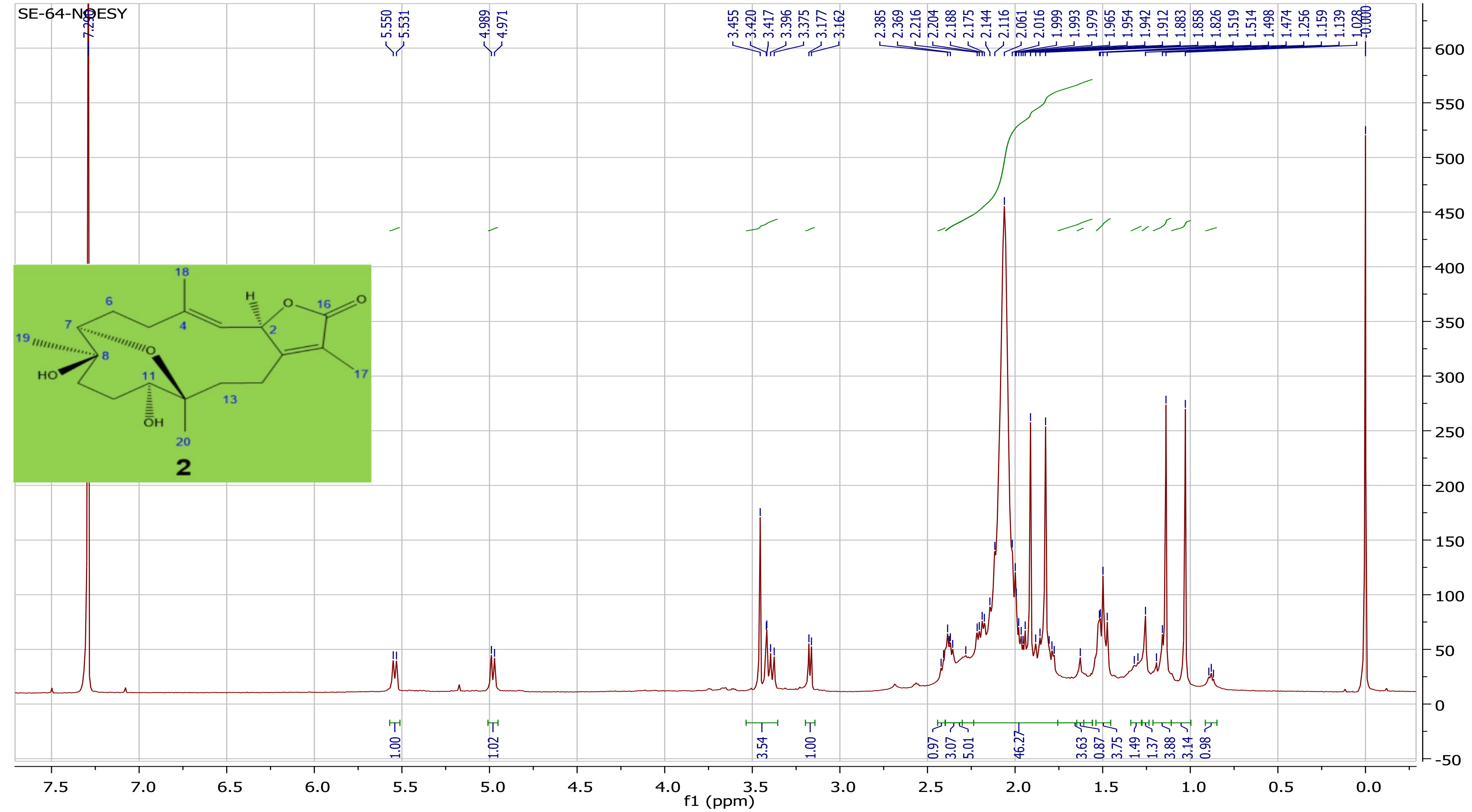

S14: ^{1}H NMR of **2**

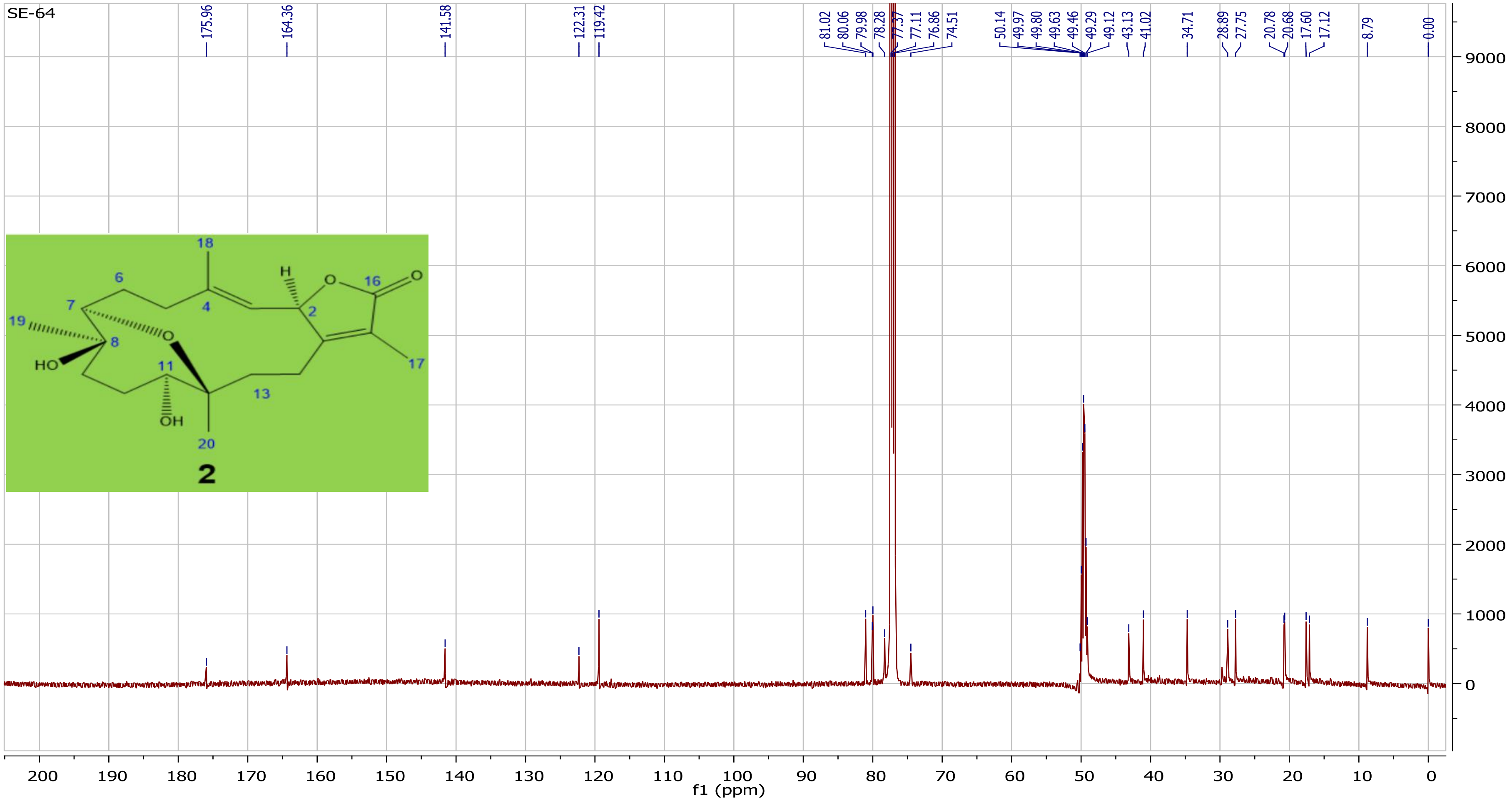

S15: ^{13}C NMR of **2**

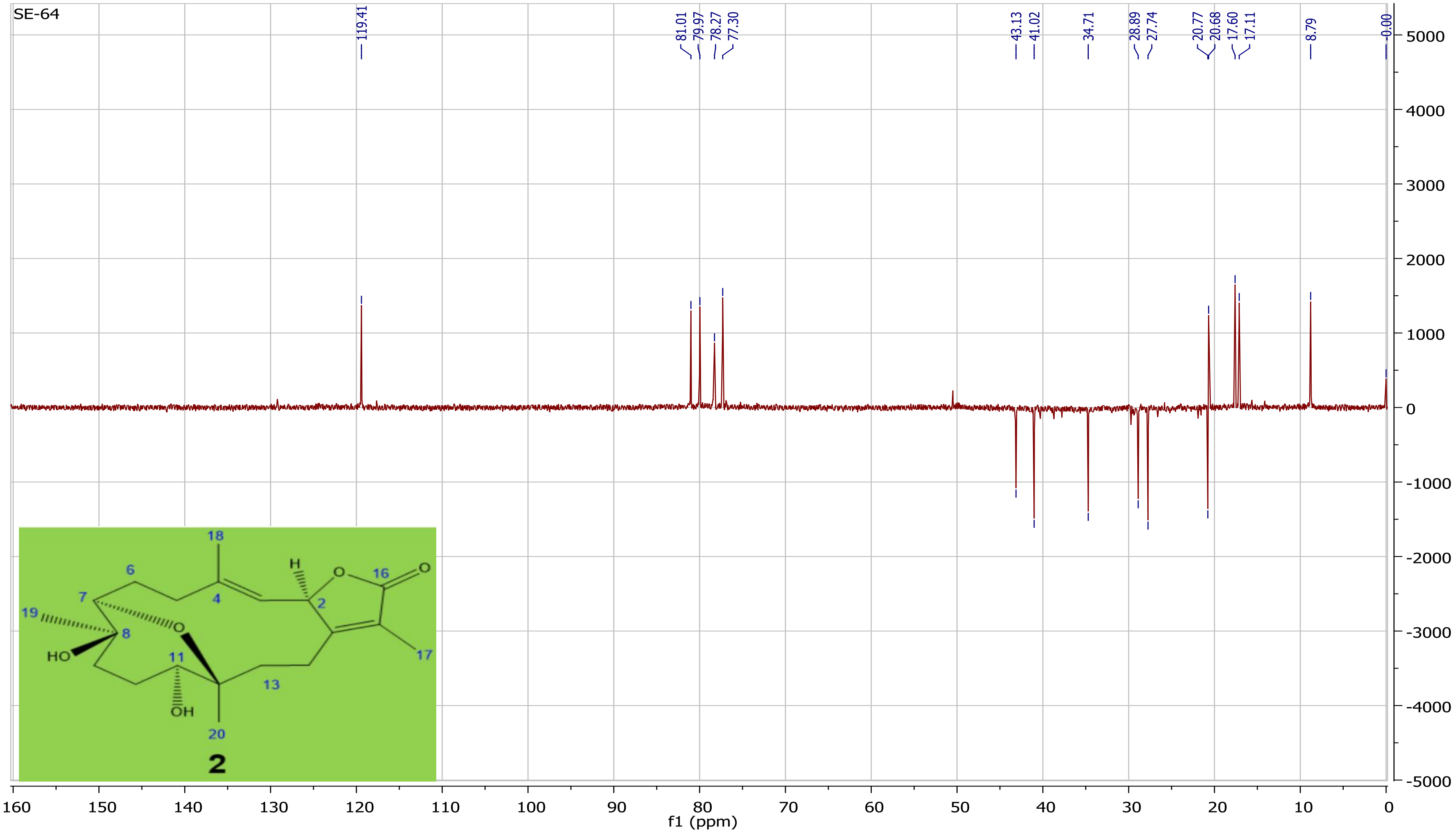

S16: DEPT-135 of **2**

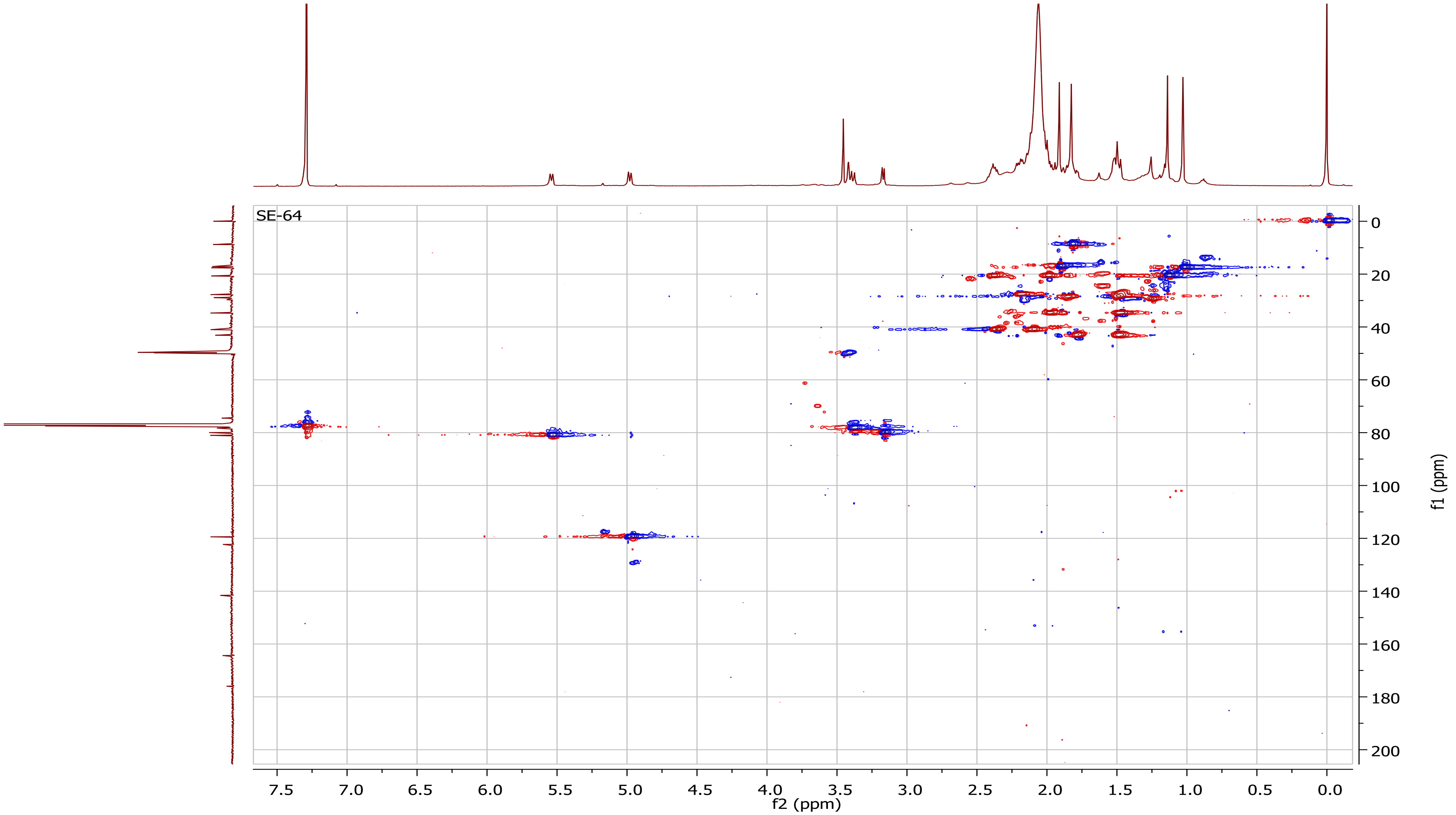

S17: HSQC of **2**

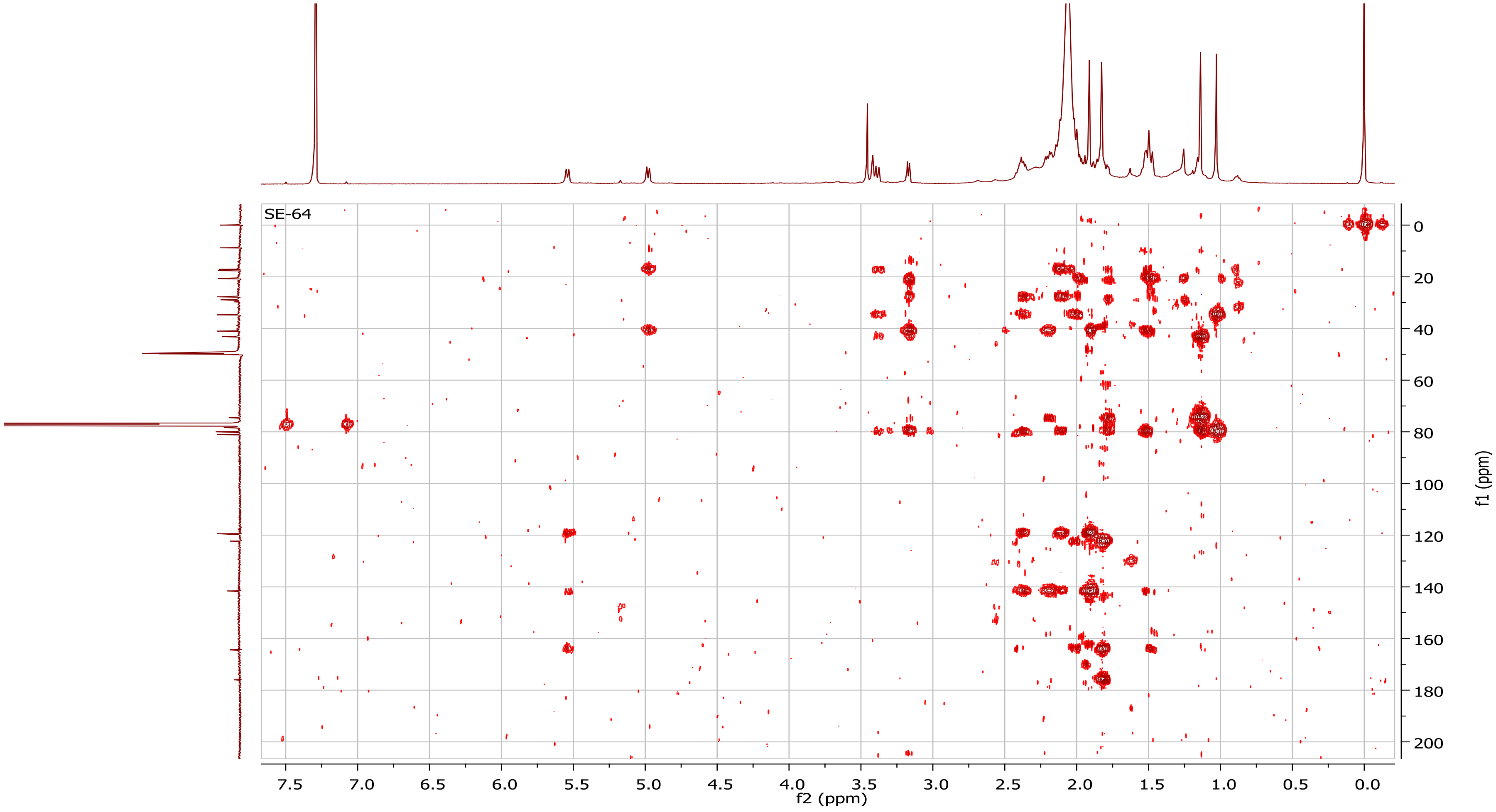

S18: HMBC of **2**

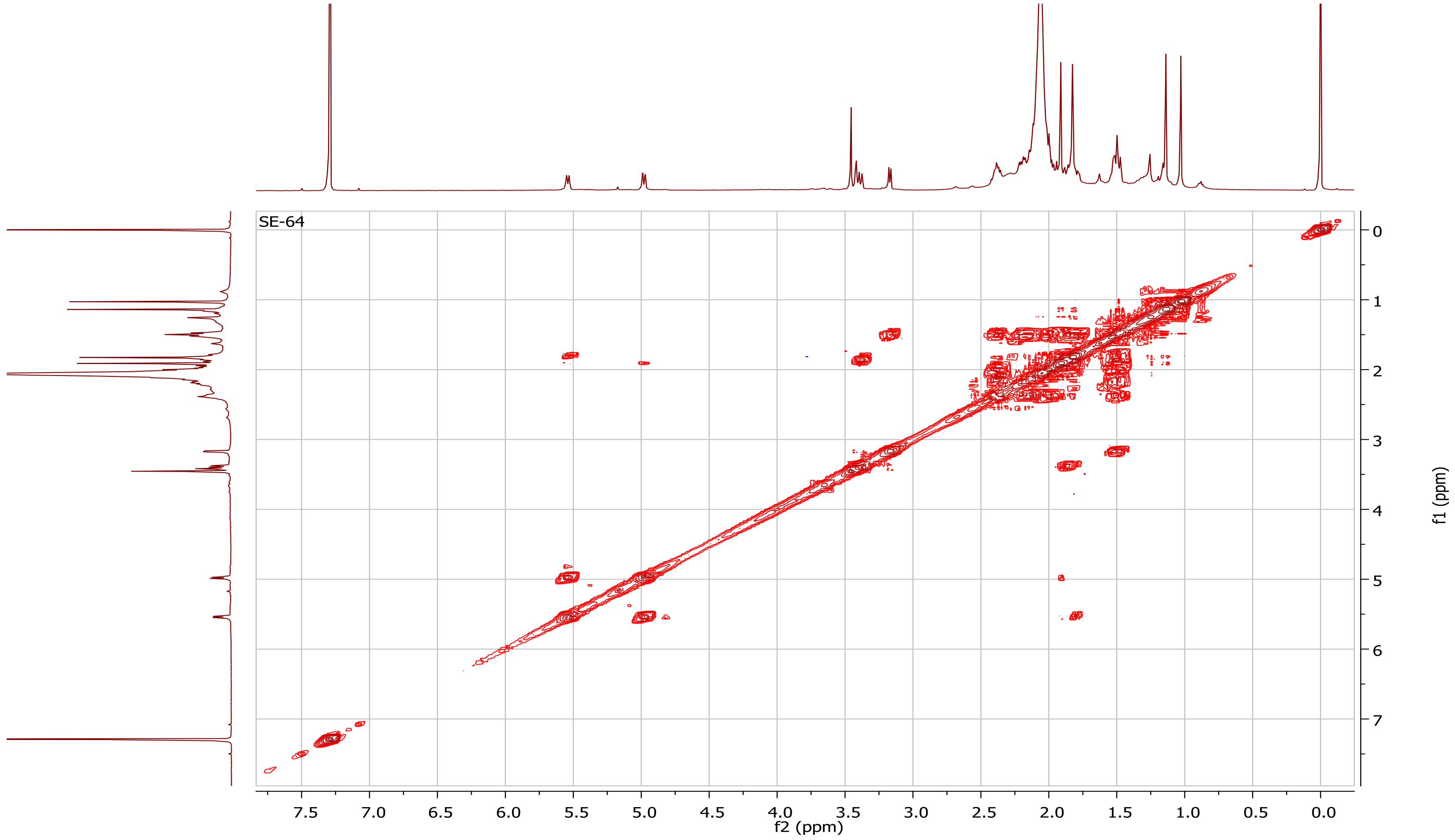

S19: $^{1}H^{1}H$ COSY of **2**

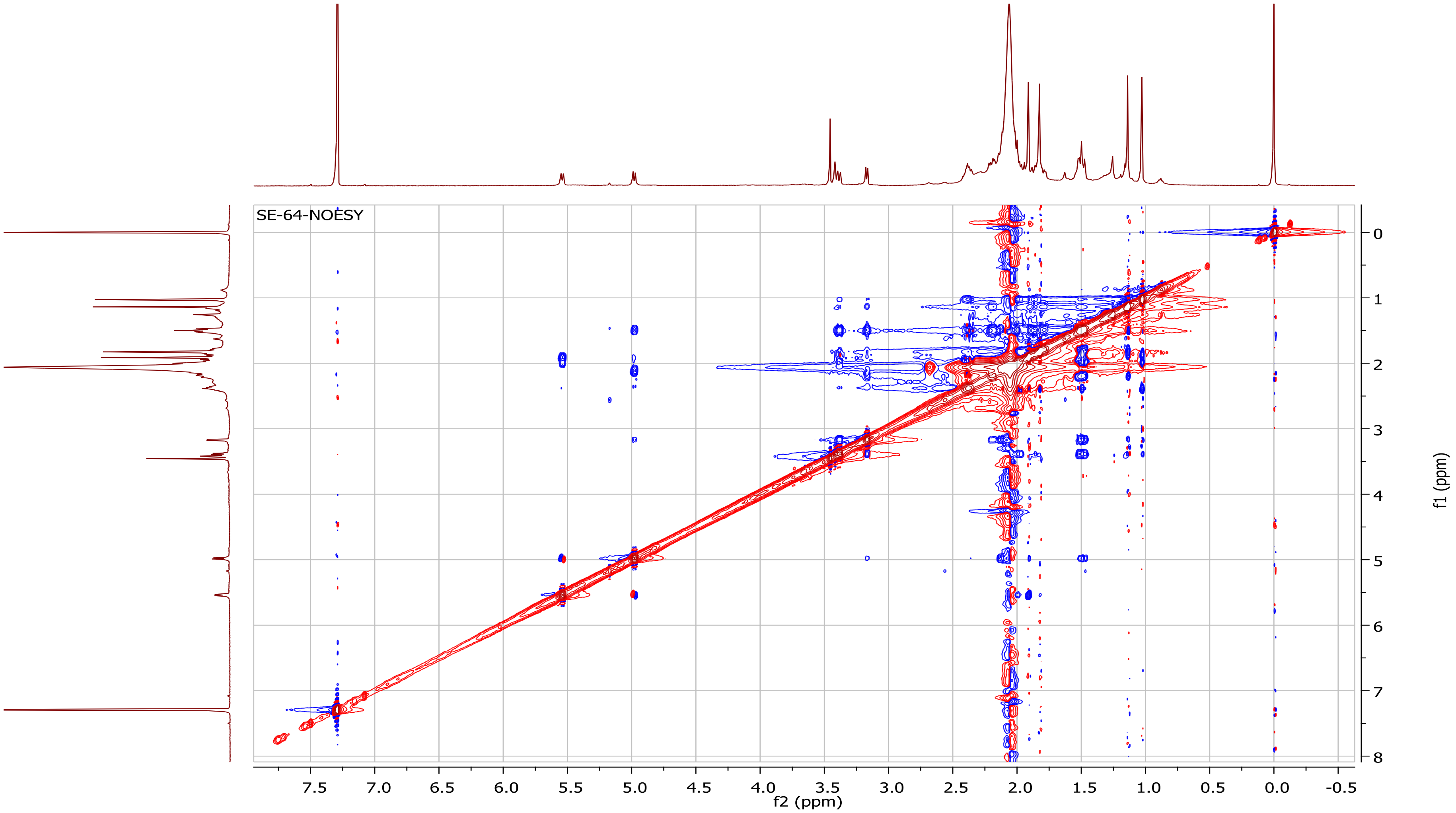

S20: NOESY of **2**

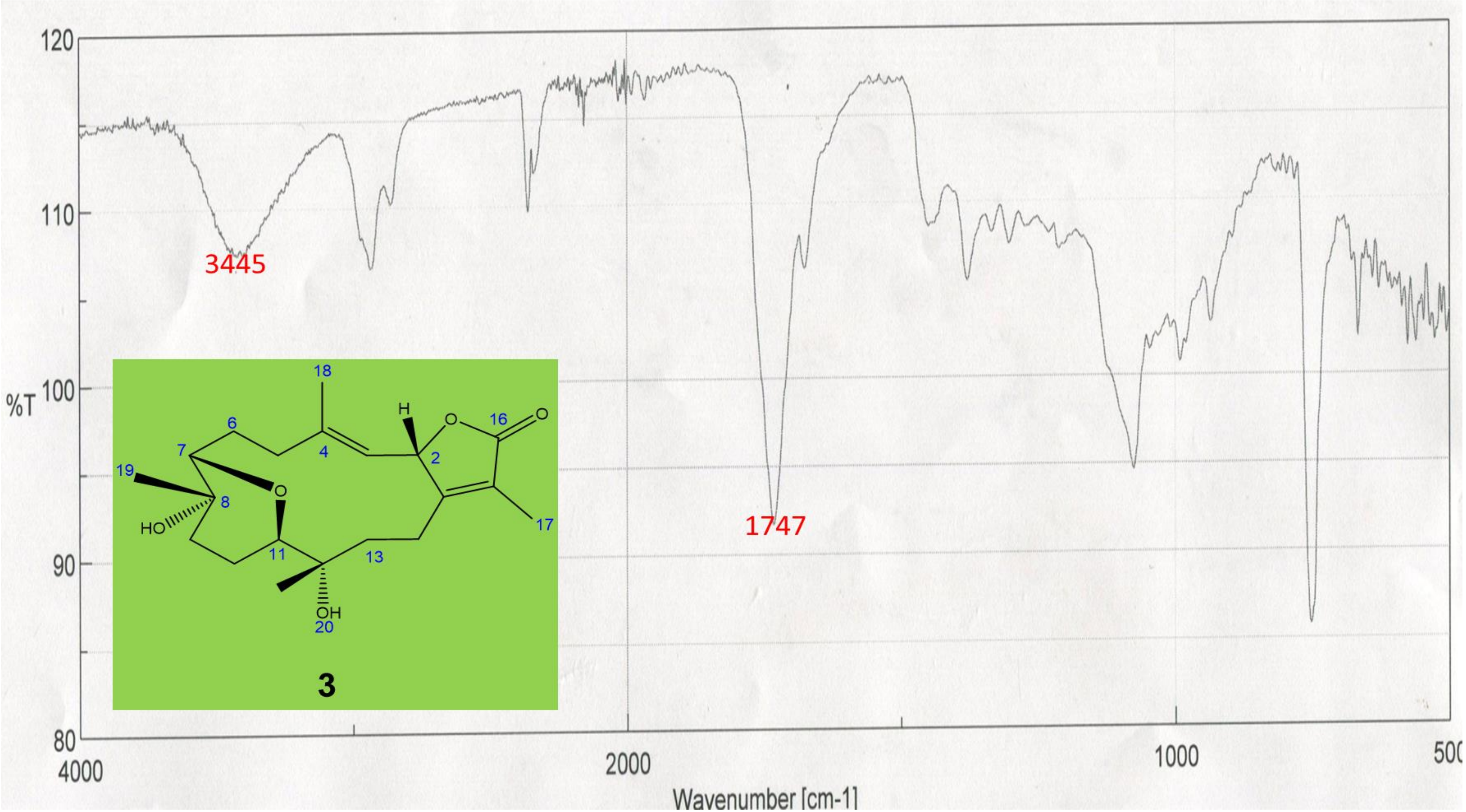
120
110
100
90
80
%T
4000
2000
1000
500
Wavenumber [cm-1]
3445
1747
18
6
7
4
H
O
16
O
2
19
O
8
HO
17
11
13
OH
20
3

S21: FT-IR of **3**

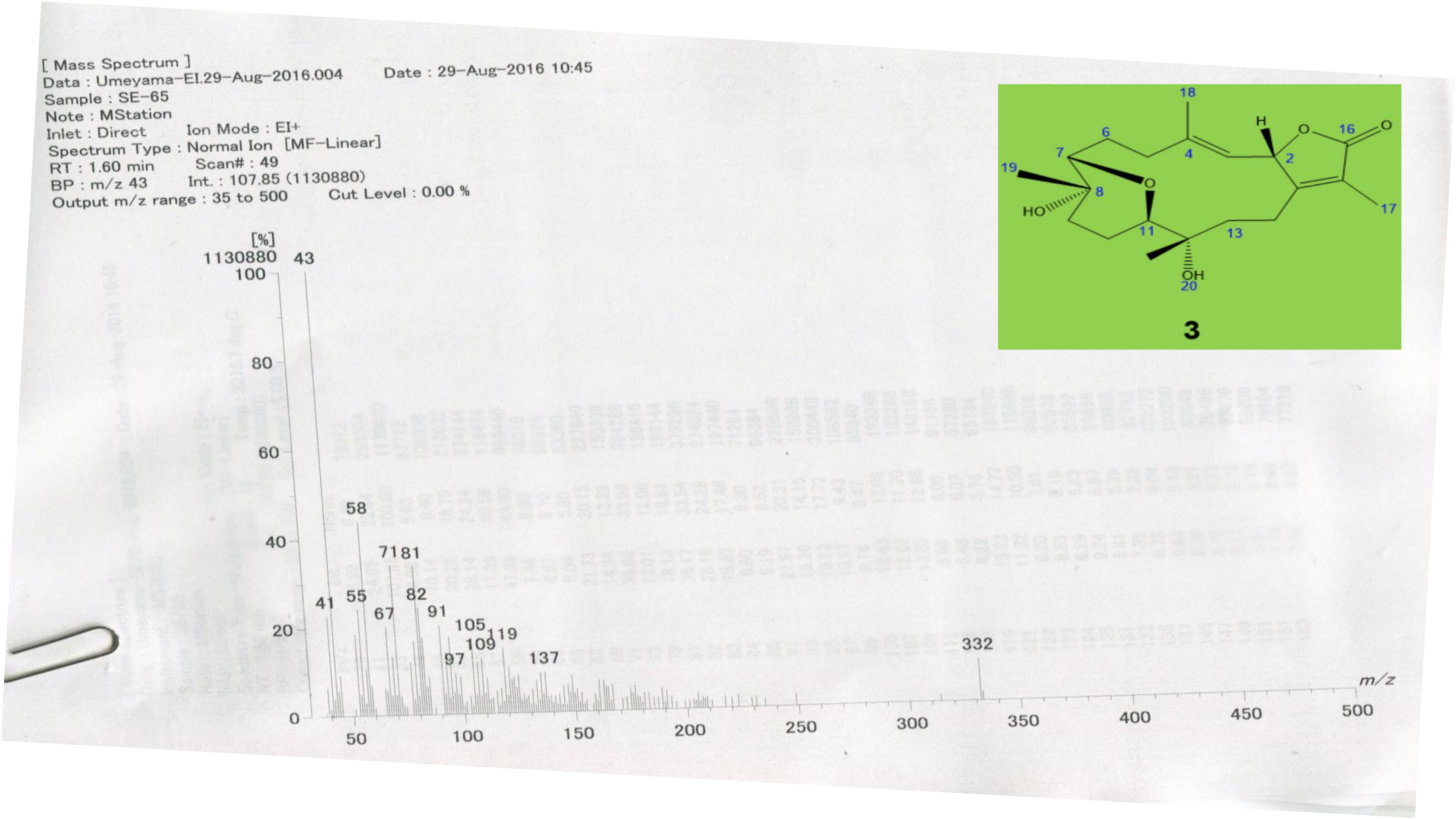

S22: LREIMS of **3**

Data : Umeyama-EIHR.29-Aug-2016.007 Date : 29-Aug-2016 14:59
Instrument : MS700D
Sample : SE-65
Note : MStation
Inlet : Direct Ion Mode : EI+
RT : 1.36 min Scan# : 35
Elements : C 150/0, H 250/0, O 50/0
Mass Tolerance : 5mmu
Unsaturation (U.S.) : 0.0 - 15.0

	Observed m/z	Int%	Err[ppm / mmu]	U.S.	Composition
1	332.1993	16.32	+1.6 / +0.5	7.0	C20 H28 O4

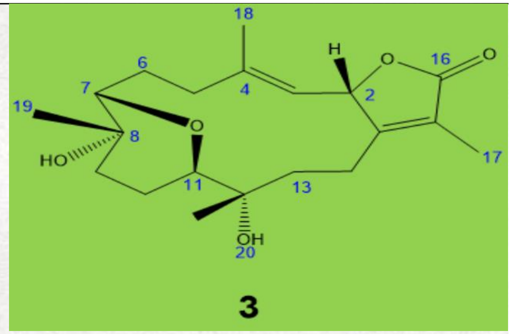

3

S23: HREIMS of **3**

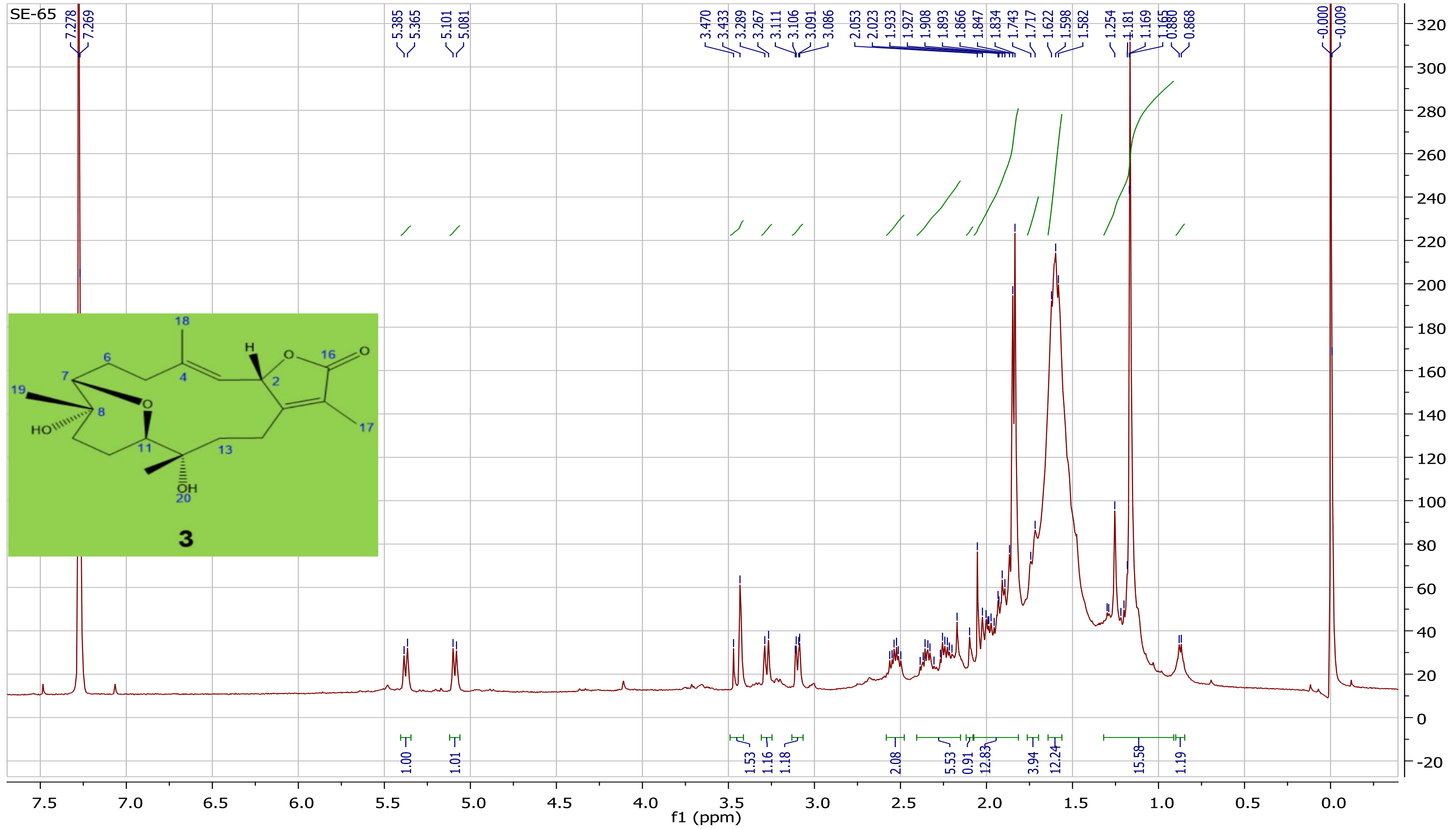

S24: ^{1}H NMR of **3**

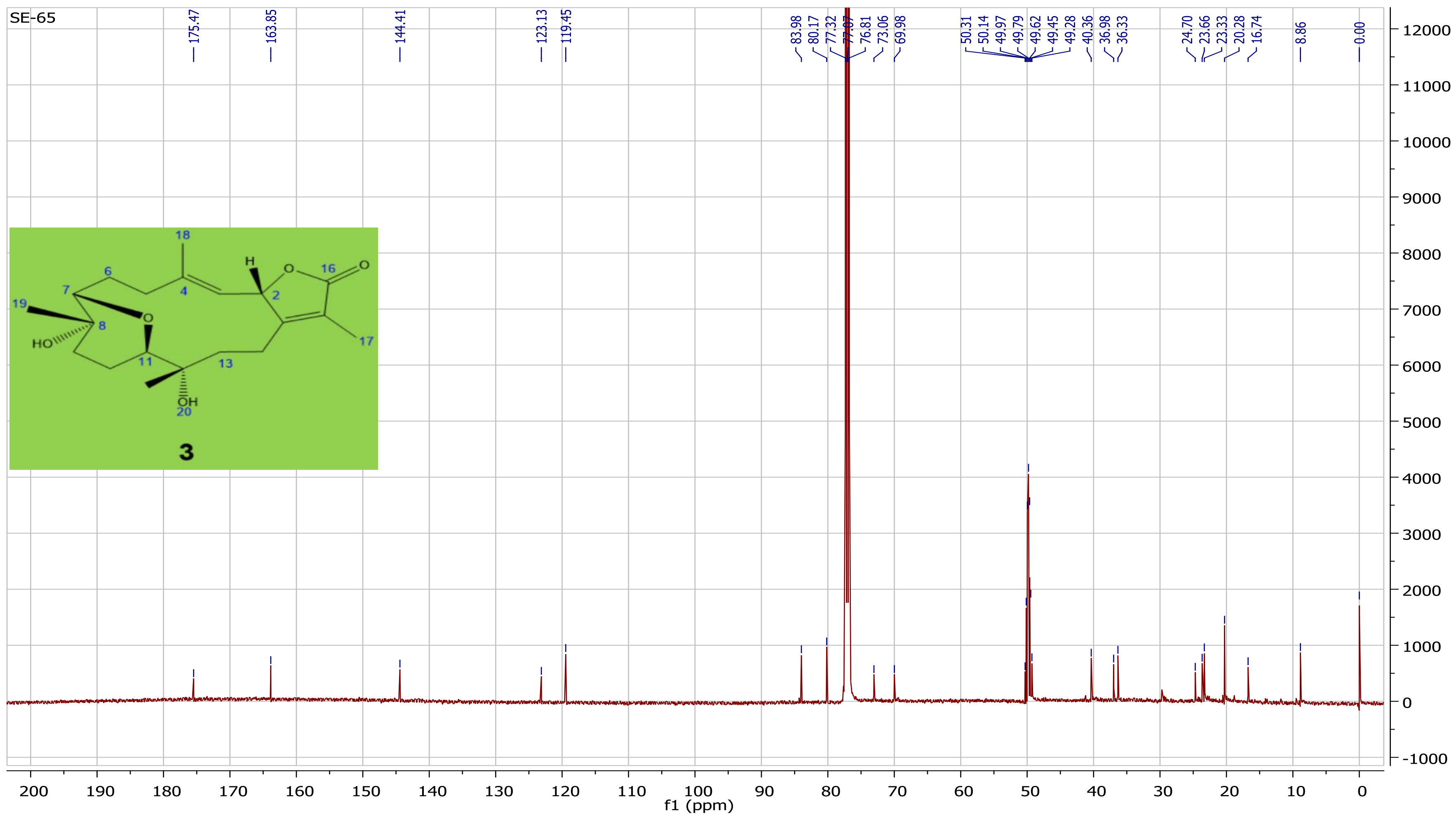

S25: ^{13}C NMR of **3**

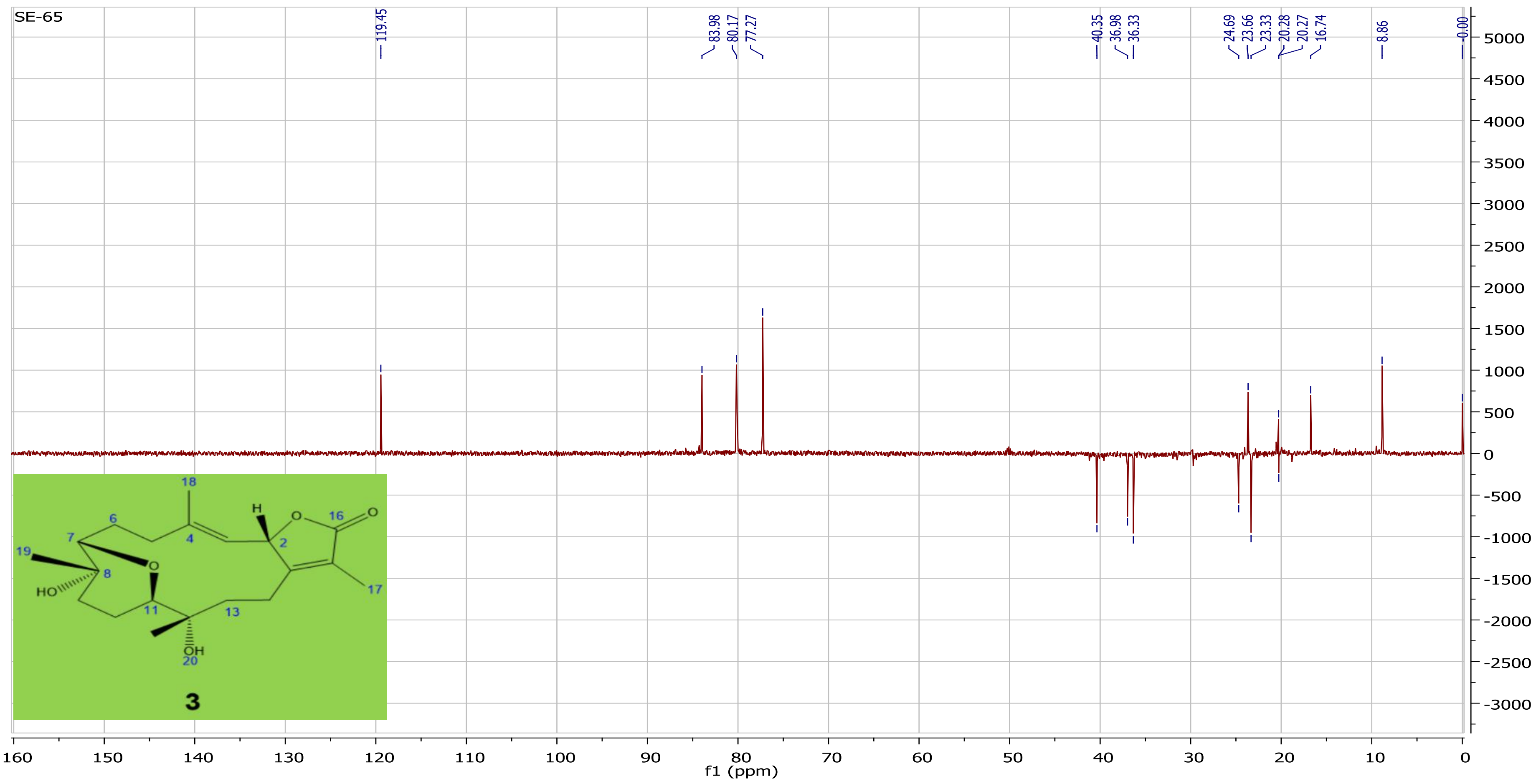

S26:DEPT-135 of **3**

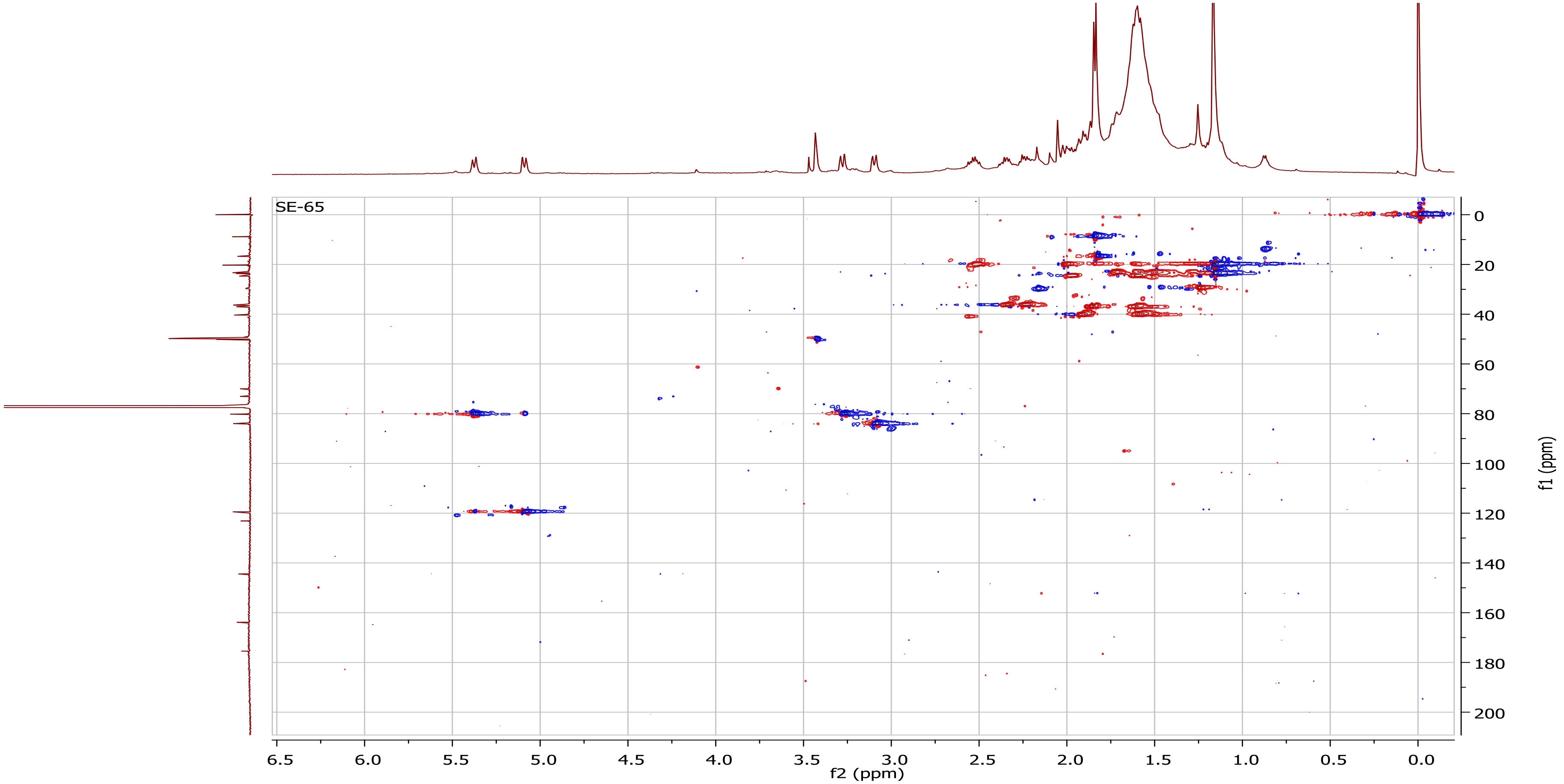

S27: HSQC of **3**

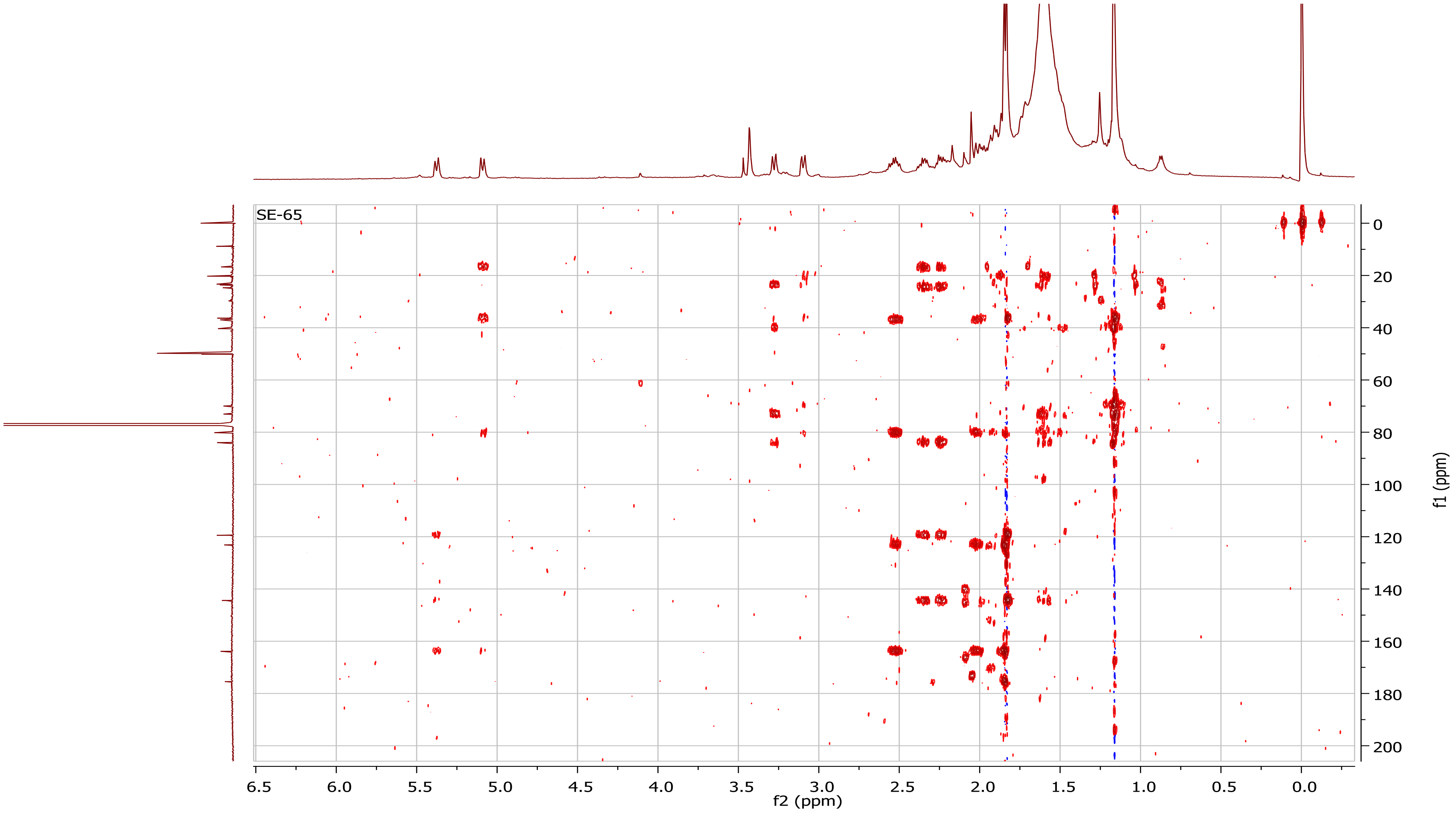
SE-65
f2 (ppm)
f1 (ppm)
6.5
6.0
5.5
5.0
4.5
4.0
3.5
3.0
2.5
2.0
1.5
1.0
0.5
0.0
0
20
40
60
80
100
120
140
160
180
200

S28: HMBC of **3**

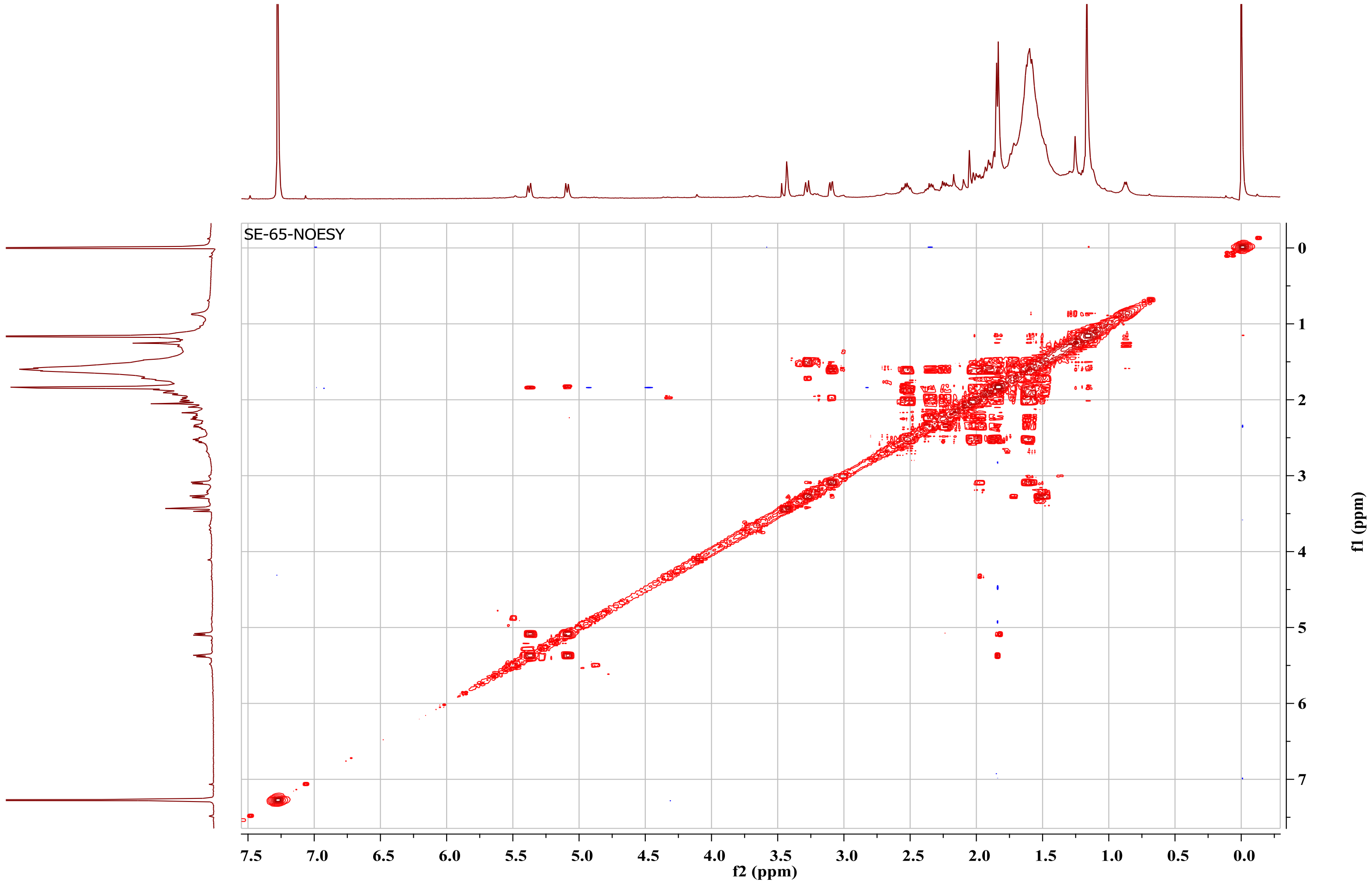

S29: ^{1}H ^{1}H COSY of **3**

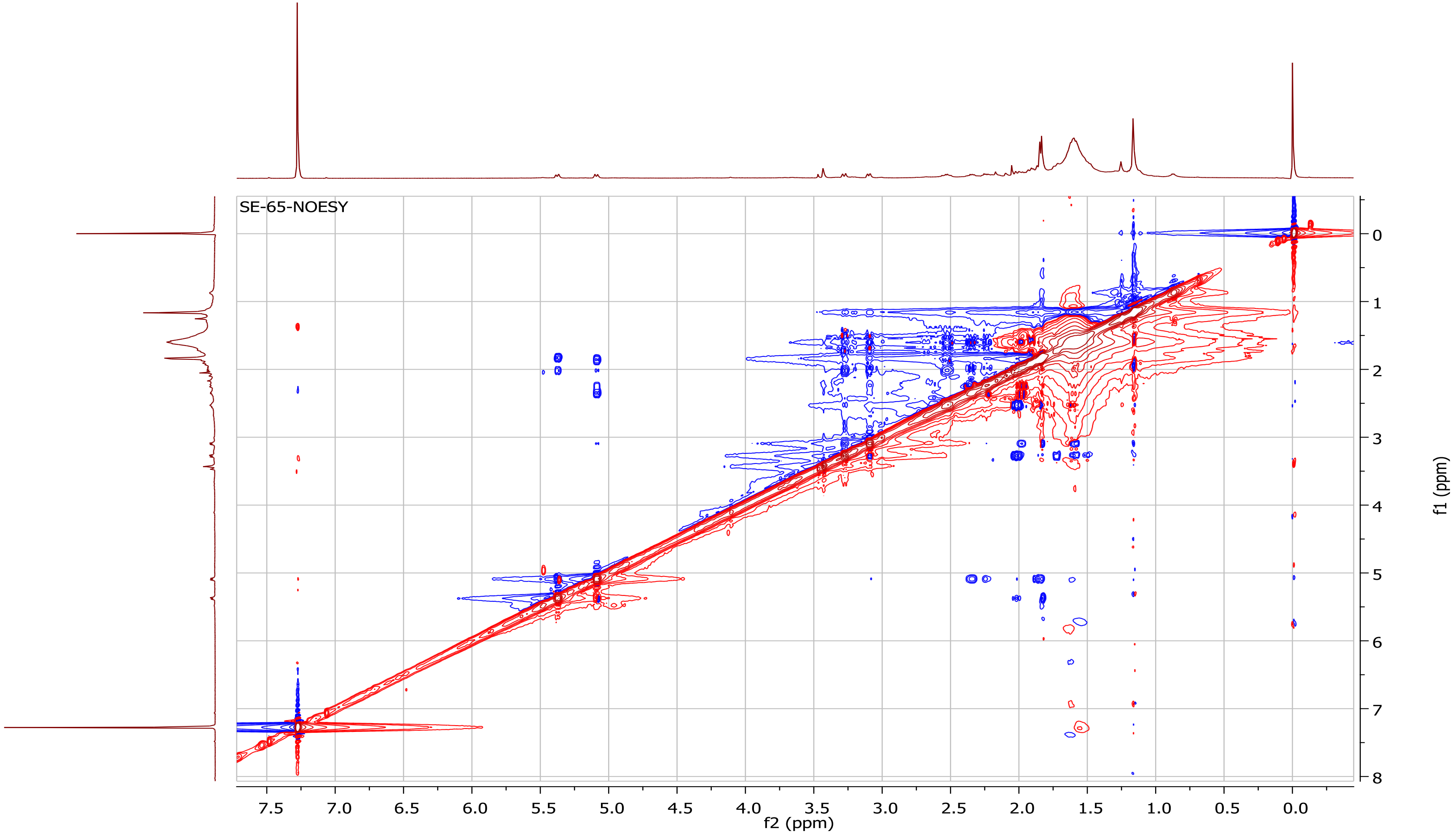

S30: NOESY of **3**
